# Birds invest wingbeats to keep a steady head and reap the ultimate benefits of flying together

**DOI:** 10.1371/journal.pbio.3000299

**Published:** 2019-06-18

**Authors:** Lucy A. Taylor, Graham K. Taylor, Ben Lambert, James A. Walker, Dora Biro, Steven J. Portugal

**Affiliations:** 1 Department of Zoology, University of Oxford, Oxford, United Kingdom; 2 Department of Infectious Disease Epidemiology, Imperial College London, London, United Kingdom; 3 School of Biological Sciences, Royal Holloway University of London, Egham, United Kingdom; Lund University, SWEDEN

## Abstract

Flapping flight is the most energetically demanding form of sustained forwards locomotion that vertebrates perform. Flock dynamics therefore have significant implications for energy expenditure. Despite this, no studies have quantified the biomechanical consequences of flying in a cluster flock or pair relative to flying solo. Here, we compared the flight characteristics of homing pigeons (*Columba livia*) flying solo and in pairs released from a site 7 km from home, using high-precision 5 Hz global positioning system (GPS) and 200 Hz tri-axial accelerometer bio-loggers. As expected, paired individuals benefitted from improved homing route accuracy, which reduced flight distance by 7% and time by 9%. However, realising these navigational gains involved substantial changes in flight kinematics and energetics. Both individuals in a pair increased their wingbeat frequency by 18% by decreasing the duration of their upstroke. This sharp increase in wingbeat frequency caused just a 3% increase in airspeed but reduced the oscillatory displacement of the body by 22%, which we hypothesise relates to an increased requirement for visual stability and manoeuvrability when flying in a flock or pair. The combination of the increase in airspeed and a higher wingbeat frequency would result in a minimum 2.2% increase in the total aerodynamic power requirements if the wingbeats were fully optimised. Overall, the enhanced navigational performance will offset any additional energetic costs as long as the metabolic power requirements are not increased above 9%. Our results demonstrate that the increases in wingbeat frequency when flying together have previously been underestimated by an order of magnitude and force reinterpretation of their mechanistic origin. We show that, for pigeons flying in pairs, two heads are better than one but keeping a steady head necessitates energetically costly kinematics.

## Abstract 2

Flocking costs may have been underestimated by an order of magnitude: a study of homing pigeons shows that they flap 20% faster when flying in pairs relative to flying solo to keep their heads steady and reap the ultimate benefits of flocking.

## Introduction

Across the animal kingdom, many species travel in groups, from pairs to flocks, shoals, herds and swarms, some containing millions of individuals [1,2]. Indeed, the collective motion of animals produces some of the most spectacular displays of synchronisation and coordination in the world [3]. Commonly cited benefits of collective travel include an improved ability to detect and avoid predators [1,4], enhanced orientational efficiency through the pooling of navigational knowledge [5–8], and energetic efficiencies derived from fluid dynamic interactions [9–13]. Flocking in birds, in particular, has received considerable attention due to the complex aerodynamic interactions that take place between group members [11–14].

Avian flock formations can be categorised as either line formations or cluster formations [15,16]. Line formations, which include the distinctive ‘V’ of many long-distance migrants, are utilised by medium- to large-sized birds, such as northern bald ibis (*Geronticus eremita*) and Canada geese (*Branta canadensis*), whereas cluster formations are typically observed in smaller birds, such as homing pigeons (*Columba livia*) and common starlings (*Sturnus vulgaris*), which fly in irregular 3-dimensional flocks [11–16]. Birds flying in close cluster flocks in particular are able to move with near perfect synchrony, whilst making rapid directional changes in 3 dimensions. Although birds travelling in V-formation can save energy by flying in aerodynamically optimal positioning within the V [11–13], those species flying in cluster flocks have been shown to incur an additional energetic cost in denser formations [14]. In homing pigeons, for example, a 10-fold increase in the spatial density of a flock has been observed to be associated with a modest 0.1 Hz increase in wingbeat frequency, which was presumed to be accompanied by an energetic cost over the 7 flights that were observed [14]. Qualitatively similar effects have been observed in flocking jackdaws (*Corvus monedula*) and rooks (*Corvus frugilegus*) [17]. Flapping flight is the most energetically demanding form of sustained forwards locomotion that vertebrates perform [18,19], and flock dynamics may therefore have significant implications for individual energy expenditure and lifetime fitness. However, no studies have yet compared the biomechanical consequences of flying in a pair to flying solo, so the energetic impact of this minimal form of flocking is unknown.

**Fig 1:**
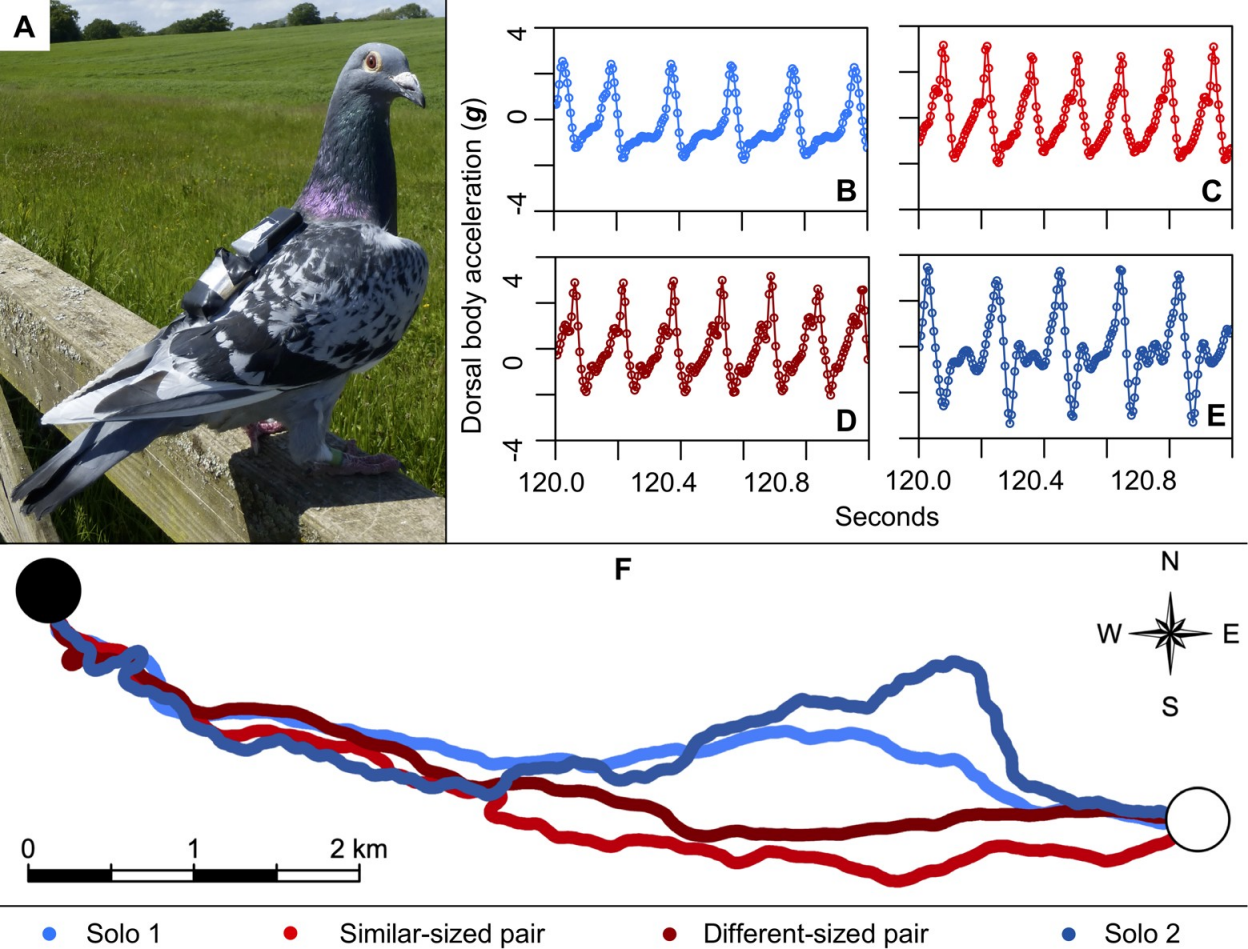
Examples of accelerometer and GPS data recorded during solo and paired flights. (A) Bird S30 carrying an accelerometer (top) and GPS sensor (bottom) attached via Velcro strips. (B–E) DB acceleration recorded by the accelerometer during S30’s final release in each of the 4 conditions: (B) solo flight (blue); (C) paired flight with a similar-sized bird (red); (D) paired flight with a different-sized bird (dark red); and (E) solo flight (dark blue). Accelerometer data have been filtered and gravity removed (see Materials and methods). Note the higher wingbeat frequency when the bird is flying in a pair. (F) Routes flown by S30 during the final release of each of the 4 conditions (same flights as those shown in Panels B–E). Note the straighter trajectory, and hence greater route accuracy, of the paired flights. Black circle corresponds to the release site and white circle corresponds to the home loft. Scale bar shows 2 km. Data deposited in the Dryad Digital Repository [23]. DB, dorsal body; GPS, global positioning system.

**Fig 2:**
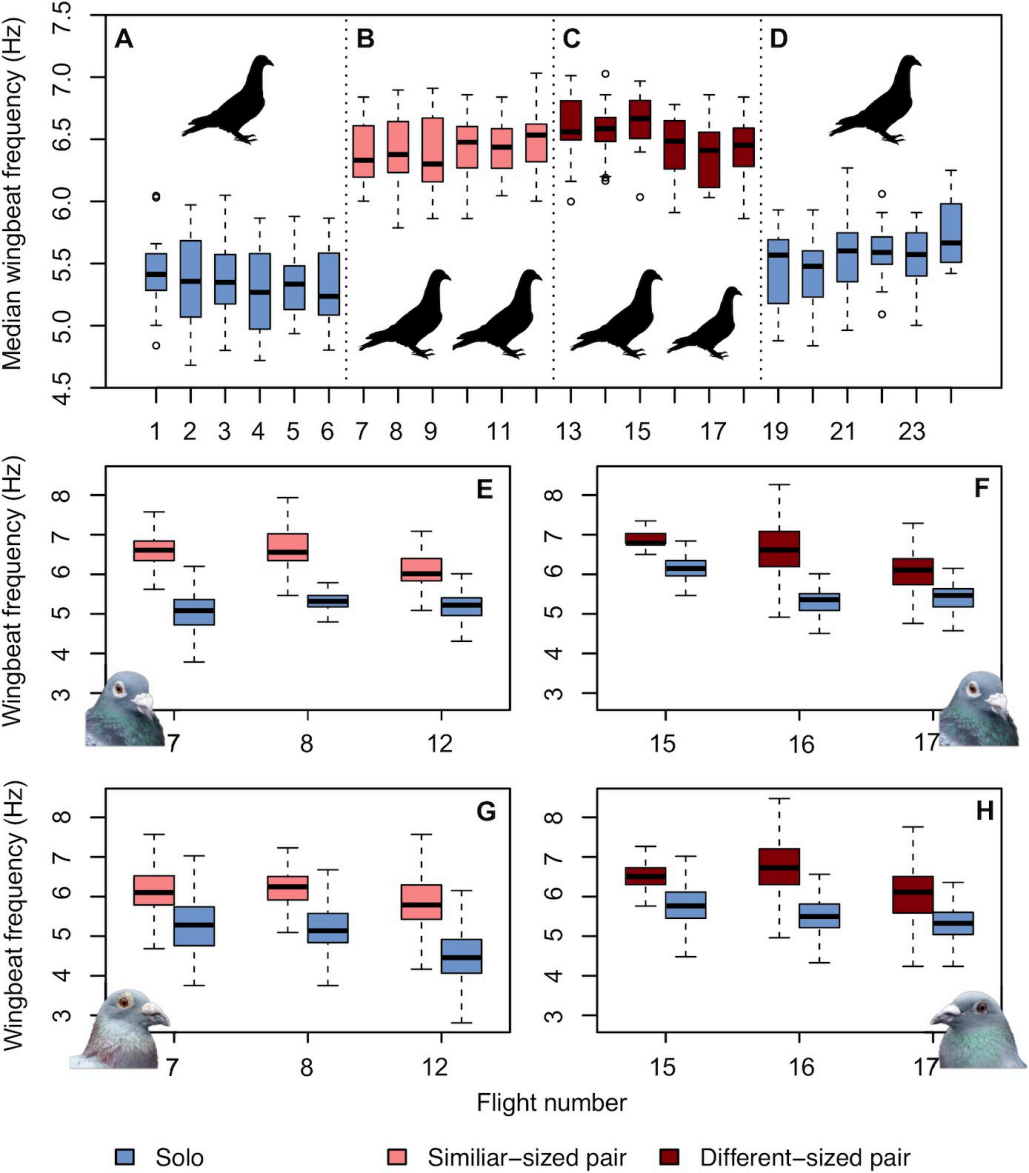
Wingbeat frequency as a function of flight number. (A–D) Median wingbeat frequency (raw data with no covariates) for all 20 birds during each of the 4 experimental phases: (A) 6 individual releases (solo); (B) 6 releases with a similar-sized bird (similar-sized pair); (C) 6 releases with a different-sized bird (different-sized pair); and (D) 6 individual releases (solo). (E–H) Raw wingbeat frequency data for birds which flew in a pair (<50 m distance) and solo (>300 m) during the same flight. (E) and (G) are from the similar-sized pair of birds B01 and B82, respectively (pictured). (F) and (H) are from the different-sized pair B01 and B07 (pictured). Box plots show the median, upper and lower quartiles, and whiskers correspond to 1.5 times the interquartile range. Data deposited in S1 Data and the Dryad Digital Repository [23].

To fill this fundamental gap, we recorded the body accelerations associated with every wingbeat of 20 free-flying homing pigeons, flying solo and in pairs, as they homed from a site 7 km east of their loft (Fig 1A). The birds were equipped with 5 Hz global positioning system (GPS) trackers and 200 Hz tri-axial accelerometer bio-loggers that allowed us to reconstruct their trajectories and wingbeat patterns during each homeward flight (see Materials and methods; Fig 1B–1F) [20,21]. The experiment consisted of 4 phases. In Phase 1, each subject first completed 21 successive solo flights, the last 6 of which provided the solo baseline (Fig 1F). In Phase 2, following the solo releases, birds were released 6 times from the same—now familiar—site but in similar-sized pairs. Pairs were assigned based on similarity in body mass and structural size as measured by tarsus length [22]. Body size and mass are strong predictors of preferred flight speeds in birds, both at the intra- and interspecific levels, with optimal flight speeds usually assumed to be those for which the cost of transport (i.e., energy expenditure per unit distance) is predicted to be at its minimum [19]. Therefore, we hypothesised that for birds of different sizes either one or both birds may have to adjust their wingbeat frequency and/or amplitude to stay together as a pair, which would represent an additional ‘hidden’ compromise cost of flying with another bird. In Phase 3, immediately following completion of the 6 similar-sized pair releases, each bird was then flown in size-mismatched pairs for a further 6 flights (different-sized pairs), again from the same site. Finally, in Phase 4, upon completion of the 6 size-mismatched flights, each bird flew 6 times solo again (Fig 2D). We compared the wingbeat characteristics of birds flying in pairs relative to flying solo to determine if pigeons alter their wingbeat characteristics when flying in a pair.

## Results

We analysed the data from all flights using Bayesian hierarchical models to account for variation due to a set of environmental covariates and the individual identity of the focal bird and, where applicable, its partner (see Materials and methods). The baseline solo means of the median wingbeat frequency and median dorsal body displacement in Phases 1 and 4 were 5.48 ± 0.19 Hz and 20.68 ± 1.17 mm (mean of the means for each individual ± SD), respectively, after accounting for the effects of airspeed, date of release, and weather variables including wind support, crosswind, temperature, humidity, and air density (Fig 2A). These baseline wingbeat frequencies are consistent with those measured previously for solo pigeon flights [21,24].

### Pigeons use fundamentally different kinematics when flying in pairs

When the same birds were flown in pairs (Phases 2 and 3), their median wingbeat frequency increased by 1.00 Hz (95% Bayesian credible interval [0.60, 1.38]) relative to flying solo (Phases 1 and 4). This represents an increase of 18.2% and was not associated with greater variability in wingbeat frequency, the standard deviation (SD) of which remained stable between solo and paired flight (2% lower SD for size-matched pairs; Fig 2B and 2C). An 18.2% increase in wingbeat frequency is greater even than the range of variation in wingbeat frequency observed in pigeons flown in a wind tunnel across a range of speeds from 6 to 20 ms^−1^ [24]. Nevertheless, this marked change in wingbeat frequency was associated with a surprisingly small (3.3%) increase in airspeed (0.63 ms^−1^, 95% credible interval [0.07, 1.19]). Likewise, the median peak-to-peak amplitude of the dorsal body acceleration was similar in both solo and paired flights (difference of 0.16*g*, where *g* is gravitational acceleration; 95% credible interval [−0.16, 0.47], where the fact that the credible interval crosses zero indicates that any difference is statistically indistinguishable from zero). The net effect of this increased wingbeat frequency and unchanged amplitude of dorsal body acceleration resulted in a net 22.5% reduction in the median peak-to-peak amplitude of dorsal body displacement (−4.65 mm, 95% credible interval [−6.21, −3.06]) through the wingbeat, as a result of the correspondingly shorter time period over which dorsal body acceleration is integrated to produce dorsal body displacement (see S1 Text for analysis of the oscillatory accelerations experienced by an accelerometer).

### The kinematics of paired flight are independent of size and leadership within the pair

These statistical findings were consistent within and between pairs, irrespective of whether the birds were flying in similar-sized or different-sized pairs (0.01 Hz increase per mm difference in tarsus length between the pair; 95% credible interval [−0.13, 0.16]; S1 Fig), or whether the bird was in front or behind during the majority of the flight (0.01 Hz increase for travelling behind, 95% credible interval [−0.10, 0.12]). In 76% of paired flights, one bird was consistently in front (>90% of all GPS points), with the remaining pairs swapping positions. The probability of whether an individual bird flew ahead in a given pair for the majority of the flight was unaffected by the birds’ tarsus length (0.01 per mm difference in tarsus length, 95% credible interval [−2.68, 2.44]), solo airspeed (−0.22 per ms^−1^ difference in median solo airspeed, 95% credible interval [−3.08, 2.65]) or body mass (0.02 per g difference in body mass, 95% credible interval [−0.10, 0.15]; S2 Fig), meaning there is no evidence that the larger or faster bird sets the pace by being in front of their partner.

In contrast to the closely coordinated flight of birds flying in V-formation [12], there was no correspondence between the front and back bird’s median wingbeat frequency (β = −0.24, where 1.0 corresponds to a direct correlation, 95% credible interval [−1.01, 0.50]; S3 Fig). It follows that their wingbeats cannot have been phase-locked for most of each flight, given that frequency matching is a necessary and sufficient condition for phase-locking of a pair of motions, and would show up as a correlation in the median wingbeat frequency within pairs if it were prevalent during each flight. All of these results were computed after accounting for the birds’ median airspeed, date of release, and weather variables, none of which had an effect on wingbeat frequency that was distinguishable from zero (S3 Fig, S1 Table). Hence, although we found no evidence in support of our hypothesis that birds of different sizes would specifically have to adjust their wingbeat frequency or amplitude to stay together as a pair, we found clear evidence that both birds in a pair increased their wingbeat frequency, independent of individual size or solo flight speed.

### Birds flying in pairs revert to solo flight kinematics if they separate

In addition to these results for all releases, one similar-sized pair and one different-sized pair separated during 3 releases each, which meant we could fortuitously compare sections of paired and solo flight within the same release. The results for these 6 releases confirm that wingbeat frequency increases as a direct result of flying in a pair, because the birds’ median wingbeat frequency decreased by 1.01 ± 0.30 Hz (mean ± SD) after they separated and flew solo (raw values with no covariates; Fig 2E–2H).

### The kinematics of paired flight depend upon spacing within the pair

As previous research has shown that pigeons increase their wingbeat frequency by up to 0.1 Hz as flock density increases [14], we analysed the effect of horizontal interindividual distance ranging from 0 m (i.e., directly above or below another bird) to 50 m (i.e., the cut-off point for flying in a pair) in a random sample of 100 wingbeats from each flight. This subsampling was necessary due to the computational demands of fitting Bayesian hierarchical models to the totality of the data. In total, we analysed 45,500 wingbeats from solo and paired flights. A Bayesian hierarchical model fitted to these data predicts that birds flying with no horizontal spacing have the highest wingbeat frequency (increase of 1.21 Hz relative to flying solo; 95% credible interval [0.81, 1.61], 21.6%), with wingbeat frequency decreasing by 0.011 Hz for every metre increase in horizontal spacing (95% credible interval [−0.012, −0.009]). Thus, birds flying 50 m apart had an expected wingbeat frequency 0.54 Hz lower than birds flying 0 m apart. Nevertheless, the act of flying in a pair still had a larger overall effect than the distance between (or density of) the birds, which meant that even birds flying 50 m apart increased their wingbeat frequency by 0.66 Hz (11.9%) relative to flying solo. Considering all of the data for all paired flights, our birds flew with a median GPS spacing 12.12 ± 4.76 m (mean of the means ± SD for each pair), which equates to a 1.07 Hz increase in wingbeat frequency under the fitted relationship (cf. the 1.00 Hz increase in median wingbeat frequency measured over the whole flight).

### The higher wingbeat frequency of paired flight is the result of a shortened upstroke

To explore the mechanism underlying the observed changes in wingbeat frequency, we divided each wingbeat into an upstroke and a downstroke phase. We defined these phases with respect to the peaks and troughs of the dorsal body acceleration, which results from a combination of aerodynamic and inertial forcing (see S1 Text for further detail). Whereas the dorsal aerodynamic force is expected to peak mid-downstroke when the wing reaches its maximum flapping speed, the dorsal inertial force is expected to peak at the start of the downstroke when the wing’s downwards acceleration is maximal. It follows that the maximum dorsal body acceleration will be reached somewhere between the start and middle of the kinematic downstroke, and similarly for the minimum, which will be reached somewhere between the start and middle of the kinematic upstroke. Hence, the downstroke phase, which we define as running from the point of maximum to minimum dorsal body acceleration, is expected to lag the kinematic downstroke slightly (and similarly for the upstroke) but by less than a quarter of a cycle. With these definitions, we found that birds reduced the median duration of the upstroke phase by 21.4% (−28.63 ms, 95% credible interval [−36.66, −20.36]) when flying in pairs, whereas the median duration of the downstroke phase did not vary significantly (−3.55 ms, 95% credible interval [−8.60, 1.67]; Fig 2C and 2D). It is clear by inspection of the wingbeat acceleration traces that this decrease in upstroke duration results in a less asymmetric pattern of forces between the 2 wingbeat phases (compare red versus blue lines in Fig 1B–1E), so that this change in wingbeat frequency essentially represents a switching of kinematic—if not aerodynamic—gait [25].

### The kinematics of paired flight entail higher mechanical power input

Aerodynamic power requirements typically display a U-shaped scaling relationship with airspeed. However, as birds cruise at airspeeds faster than their minimum power speed, their aerodynamic power requirements are expected to increase monotonically with airspeed in cruising flight. It follows that the small increase in airspeed that we observed in paired flight is expected to involve a higher mechanical input than in solo flight: a conclusion corroborated by the accompanying increase in wingbeat frequency [14,19]. A bird’s aerodynamic power requirements are classically partitioned into 3 components: a parasite power requirement, supplying the energy needed to overcome drag losses on the body; a profile power requirement, supplying the energy needed to overcome drag losses on the wings; and an induced power requirement, supplying the energy needed to accelerate the air. Since body drag varies as *D*_*b*_ = *ρU*^2^*S*_*b*_*C*_*D*_/2, where *ρ* is air density, *U* is airspeed, *S*_*b*_ is body frontal area, and $C_{D_{b}}$ is body drag coefficient, it follows that parasite power requirements scale as *D*_*b*_*U*~*U*^3^. A 3.3% increase in airspeed (95% credible interval [0.4%, 6.1%]) would therefore be expected to result in a 10.2% higher parasite power requirement in paired flight (95% credible interval [1.2%, 19.6%]).

Profile power and induced power requirements are harder to assess, but under the classical model of aerodynamic power requirements in birds [26], profile power is assumed to be approximately constant, whereas induced power is assumed to scale as *U*^−1^. This decreasing induced power requirement should partially offset the increasing parasite power requirement at speeds above the minimum power speed. In fact, a straightforward application of this classical theory predicts that a 3.3% increase in airspeed in paired flight should be associated with a small (2.9%) and only just significant (corresponding 95% credible interval [0.3%, 5.6%]) increase in the total aerodynamic power requirement. In light of the greater distance covered per unit time, this increase in aerodynamic power requirement would actually be expected to result in a small (0.4%) net saving in the cost of transport, although the effect is barely significant (95% credible interval [0.1%, 0.5%]).

This classical approach neglects the aerodynamics of flapping force production and hence the effect of wingbeat frequency, which have recently been addressed by theory [27] modelling the optimal vortex wake for a given wingbeat frequency. Not unlike the classical fixed-wing theory [26], this vortex wake theory [27] predicts that the observed 3.3% increase in airspeed and 18.2% increase in wingbeat frequency should be associated with only a 2.2% increase in the total aerodynamic power requirements in paired flight. Even treating the assumptions of the modelling as being accurate, there remains considerable uncertainty in the model estimates, because the most extreme combinations of the 95% credible intervals for airspeed and frequency lead to a credible interval of [−3.2%, 8.2%] for the predicted change in power requirements. Nevertheless, the overall expectation of a 2.2% increase in aerodynamic power implies that 13.5% less work would have to be done per wingbeat for consistency with the observed 18.2% increase in wingbeat frequency. Of course, the optimal vortex wake model explicitly assumes that the kinematics are optimised so as to minimise the aerodynamic power requirement. Hence, if the increased wingbeat frequency were not accompanied by the other changes in wing kinematics needed to produce an optimal vortex wake, then the actual increase in the total aerodynamic power requirements could be much higher—even up to 18% if the work done on each stroke remained constant. Either way, it is clear that the birds selected a substantially different kinematic gait when flying in pairs, adding approximately one more wingbeat every second, and that they did so for almost negligible gain in airspeed. As managing energy expenditure is key to survival, any additional cost of flying in a pair must be outweighed by some other benefit of flying with conspecifics.

### Paired flight provides improved homing accuracy

One key benefit commonly ascribed to flocking is the ability to pool navigational knowledge. Because this is expected to improve homing accuracy [7,8], we calculated our birds’ route accuracy flying solo and in pairs, using a weighted mean cosine of the angle between the birds’ heading and destination. Flying in a pair resulted in a 7% increase in route accuracy relative to both the Phase 1 and Phase 4 solo releases (0.06, 95% credible interval [0.01, 0.10]; Fig 1), and a concomitant 6% decrease in route length (S2 Table). This improved homing accuracy when flying in pairs is consistent with previous empirical studies [5,6] and with theoretical expectations [7,8] but presumably requires each member of the pair to attend closely to the other. This will in turn require a high degree of visual stability, and we therefore hypothesise (and, in the next section, test) that a potential function of the increased wingbeat frequency and decreased oscillatory displacement of the body in paired flight is to enhance visual stability when attending to nearby conspecifics.

### Increased wingbeat frequency is associated with decreased head displacement in paired flight

**Fig 3:**
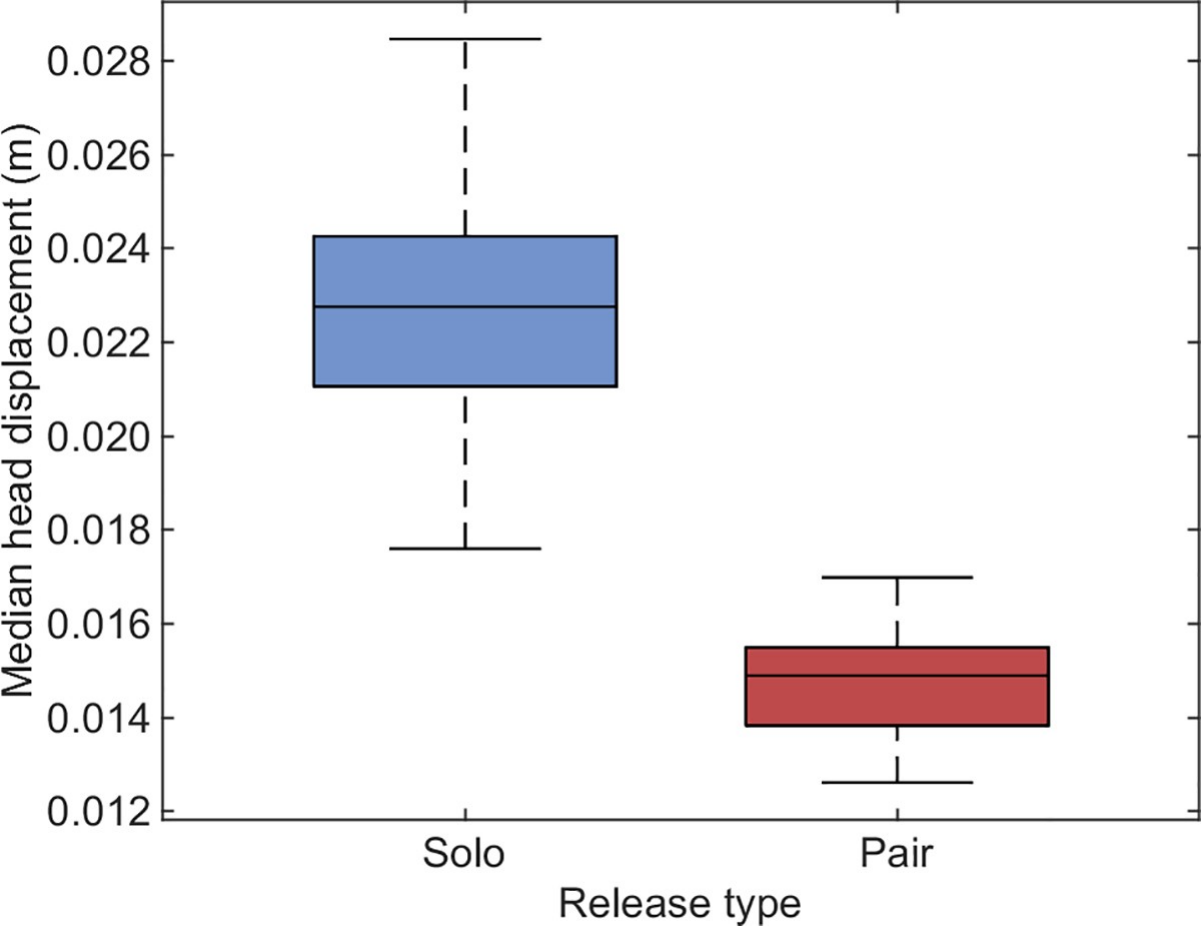
Box plot of median vertical peak-to-peak head displacement across 6 birds, each flying once solo and once in a pair. Bottom and top edges of the box indicate the 25th and 75th percentiles, and the whiskers extend to the most extreme data points. Note that there is no overlap between the median head displacements in the 2 conditions. Data deposited in the Dryad Digital Repository [23].

To test directly whether visual stability is enhanced in paired flight, we conducted a second experiment using head-mounted accelerometers on 6 homing pigeons on short-range flights (950 m), flying solo and in pairs (see Materials and methods). In close agreement with the first experiment, birds flying in pairs increased their median wingbeat frequency by a mean of 1.10 Hz ± 0.26 relative to flying solo (6.6 ± 0.42 Hz mean ± SD for pairs; 5.5 ± 0.46 Hz for solo). More importantly, however, our results also show that the median peak-to-peak head displacement simultaneously decreased by 5.3 × 10^−3^ m ± 6.6 × 10^−4^ m between solo and paired flight, representing a 30% reduction in the amplitude of oscillatory head displacement and the retinal slip this causes (Fig 3). This improvement in translational head stability will directly reduce the retinal slip of nearby objects for which motion parallax is significant (e.g., for a pair of birds flying at 1 m spacing, the motion parallax associated with the oscillatory head displacement is of order 1°). Any improvement in translational head stability is expected to be associated with a corresponding improvement in rotational head stability, which will reduce the retinal slip of objects at any distance.

### Summary

In summary, birds flying in a pair increased their wingbeat frequency by 18%, thereby accommodating one additional wingbeat per second, whilst maintaining approximately the same peak-to-peak dorsal body acceleration. They achieved this through a typical 21% reduction in the duration of their upstroke, leading to only a 3% increase in airspeed, but attenuating the amplitude of oscillatory head displacement by 30%. Relatively smaller increases in wingbeat frequency were observed when the birds in a pair flew further apart.

## Discussion

One of the most commonly cited benefits of flying together in groups is to save energy by reducing aerodynamic power requirements [11,12]. However, favourable aerodynamic interactions are only expected to arise in specific flight formations, and they may be absent or unfavourable in other flock configurations. Previous research on pigeons has in fact identified a small energetic cost to cluster flocking, which increased slightly with increasing flock density [14]. However, this study did not compare flight in a cluster-flock relative to the alternative of flying solo and inferred the increased power requirement from an increase in wingbeat frequency that was an order of magnitude smaller than we have measured for paired versus solo flight. Specifically, whereas pigeons flying in large cluster flocks increased their wingbeat frequency by 0.1 Hz across a range of increasing flock densities [14], our results show that the very act of flying with another bird increases a pigeon's wingbeat frequency by 1.0 Hz. A more recent study of flocking corvids [17] also found an increase in wingbeat frequency in large flocks, but compared this to data pooled from birds flying solo or in pairs, leaving open the question of how their wingbeat frequency would have changed relative to an unambiguous base line condition of solo flights.

The wingbeat frequencies that Usherwood and colleagues [14] measured for pigeons flying in flocks (6.6–7.0 Hz) were actually somewhat faster than those we measured in paired flight (6.5 ± 0.2 Hz) and substantially faster than those we measured in solo flight (5.5 ± 0.2 Hz). In hindsight, it is clear therefore that the wingbeat frequencies reported in flocks by Usherwood and colleagues [14] were already elevated well above those typical of solo flight, implying that this previous work would have understated the increased wingbeat frequency—and potentially also the energetic cost—of flocking by an order of magnitude. Most importantly, our observation that pigeons increase their wingbeat frequency by 18% when flying in pairs relative to flying solo suggests that the majority of this effect comes from the mere act of flying with another individual, rather than from the density of the flock. Nevertheless, we did find that variation in interindividual spacing had a significant effect on wingbeat frequency over an extended range of distance, such that birds flying 50 m apart would have had an expected wingbeat frequency 0.54 Hz lower than birds flying at 0 m spacing. What explanation could there be for this spacing effect, given that any aerodynamic interactions between the birds must occur on spatial scales many times smaller than those over which an effect of spacing was observed?

Two key hypotheses have been proposed for the increase in wingbeat frequency seen in denser cluster flocks: (i) negative aerodynamic interactions between flock members and (ii) increased need for control and collision avoidance [14]. Whereas theoretical work investigating induced drag and a small effect of spacing within a flock led previous work to hypothesise a possible aerodynamic basis to the costs of cluster flocking [14,28], our work clearly demonstrates that both birds within a pair increase their wingbeat frequency, which indicates these effects are unlikely to have been related to negative aerodynamic flow interactions, because the bird in front does not fly in the wake of the bird behind. Indeed, whether each focal bird was in front or behind had almost no effect on its measured wing kinematics, which is not what we would expect if the effect of flying in pairs arose from their aerodynamic interaction. Likewise, there was no effect of the size of each focal bird relative to its partner. It therefore seems unlikely that aerodynamic interactions between individuals could be the ultimate cause of the substantial increase in wingbeat frequency observed in birds flying in pairs or larger flocks.

Instead, we hypothesise that birds increase their wingbeat frequency when flying together so as to enhance their manoeuvrability and flight stability [14,29–31]. Birds flying in a flock need to manoeuvre quickly and continuously to remain together and avoid collisions [14], which will be especially important when flying in close proximity to each other. Since birds make their main kinematic control inputs on a wingbeat-to-wingbeat basis in flapping flight, elevating wingbeat frequency will increase the rate at which discrete control inputs can be applied, thereby enhancing the ability to respond to the movements of others. Flocking birds also require a high degree of visual stability to observe and coordinate with other individuals. This holds true at any distance but will be most important when birds fly close enough together that the effects of motion parallax are significant [32,33]. Indeed, pigeons flying in pairs have already been found to reduce the frequency of angular head saccades relative to flying solo, which suggests either an increased focus on their partner or a decreased focus on the environment [34]. Increasing a bird’s wingbeat frequency is expected to amplify any inherent flight stability that the bird may already have [31], which will enhance visual stability by damping the effects of any external perturbations at the level of the whole bird. Moreover, as we have shown both in theory (see S1 Text) and in practice (see Results), a straightforward 18% increase in wingbeat frequency automatically reduces the oscillatory displacement of the head by approximately 30% during flapping.

Birds have a limited range of eye movement, and visual stabilisation is therefore facilitated by compensatory motions of the neck motor system, mediated by visual, vestibular, and proprioceptive cues [35,36]. Without these image stabilisation mechanisms, birds would have difficulty in differentiating the motion of a target or obstacle from their own head or body motions, which is especially problematic when viewing nearby objects or conspecifics. Passive head stabilisation mechanisms can also be important, with a high degree of viscoelastic damping provided by the neck itself [37], particularly in species with long necks. It is interesting to note that long necks are typical of large flocking birds with low wingbeat frequencies, such as swans and geese (Anatidae), pelicans (Pelecanidae), ibis (Threskiornithidae), and cranes (Gruidae). These species will not benefit from the low head displacement amplitudes that result at high wingbeat frequency, so for them a long neck may be essential to providing the visual stability needed to enable coordinated flocking. This enhanced neck stability perhaps explains why pelicans flying in V-formations are able to do so whilst displaying a small reduction in wingbeat frequency associated with the aerodynamic benefits of flocking [11]. Conversely, elevated wingbeat frequency may be particularly important in stabilising vision in short-necked birds, which will not benefit from the degree of damping that is provided by having a long neck [37].

Managing energy expenditure is critical for survival and is a primary focus for natural selection. Aside from their directly observed changes in wing kinematics, birds flying in pairs are expected to have a higher aerodynamic power requirement than birds flying solo on account of their higher airspeed. Concurrently, birds flying in pairs were able to improve route accuracy by 7%, taking 9% less time on average to return to the loft, despite flying a familiar route. Birds flying in pairs will therefore experience a net saving in total energy consumption provided that their metabolic power requirements are not increased by more than 9% in paired flight. On the assumption that the wingbeat kinematics are fully optimised in relation to each flight condition, the total aerodynamic power requirement is estimated to be approximately 2% higher in paired than solo flight. If the wingbeat kinematics are not fully optimised, then the increase in aerodynamic power requirement could be rather higher, though probably by less than the 18% that would be implied by the increase in wingbeat frequency if the work per stroke were held constant. Despite this, only 6 releases had to be repeated due to birds separating at the start (5%), and only 12 (10%) of those 116 pair releases in which birds did not separate at the start resulted in separation later on during the flight. Hence, the observed preference for paired flight suggests that either (i) the general strategy of flocking is adaptive because the navigational benefits of flocking are sufficient to outweigh the increased cost of transport, or (ii) the other benefits of flocking, such as predator protection, outweigh the increase in energy expenditure required to fly in pairs, even over quite short distances.

Although minimizing energy consumption may not be an especially strong selection pressure for homing pigeons that have been selectively bred to return quickly to the loft, and which have ad libitum access to feed, the results of our study nevertheless indicate that flying with conspecifics entails an energetically expensive alteration to wingbeat kinematics. As many other species of birds also preferentially fly in cluster flocks, our results suggest that the additional benefits of flocking must outweigh any accompanying increase in aerodynamic power requirements. The overall 9% reduction in homing flight time that we observed represents a 9% reduction in the period over which our birds were exposed to predation risk when returning to the loft. Moreover, not only does the act of flying in a pair dilute the chance of fatality during a predation event by 50%, but the probability that such a predation event is successful decreases as flock size increases, presumably through a combination of increased opportunity for vigilance and predator confusion effects [38]. Therefore, for pigeons, the ultimate benefits of flying together, such as protection from predators and the pooling of navigational knowledge, must together outweigh the increased aerodynamic power requirement of flying with conspecifics.

Overall, the results of our study of a cluster-flocking species stand in contrast to previous studies of birds flying in V-formations, which have provided clear evidence for aerodynamic benefits to flocking [11–13], including in one case an actual reduction in wingbeat frequency [11]. Formation flight is typically utilised by medium- to large-sized birds during goal-orientated movement, whereas cluster-flock formations are utilised by smaller birds, such as starlings, in movements ranging from oriented to highly tortuous motion [15,16]. Bird size and the complexity of their movement paths may both contribute to the observed differences in wingbeat patterns between flock formation types. Whilst birds flying in V-formations are able to fly in aerodynamically optimal positions to conserve energy, the naturally higher wingbeat frequencies of smaller birds, their smaller turning angles, and the rapidity of their directional changes may preclude flying in energy-saving formations and instead necessitate a wingbeat pattern that facilitates a greater degree of control. Thus, the demands of moving together in an irregular 3-dimensional flock or pair are sufficient to alter the very way in which a bird flies. Taken together, our results demonstrate that pigeons modify their wing kinematics substantially when flying in pairs, leading to an overall increase in their aerodynamic power requirement, rather than the decrease observed in pelicans [11]. The fact that they do this implies that, for pigeons, the act of flocking is both fundamentally important and fundamentally expensive.

## Materials and methods

### Experiment 1

#### Ethics statement

The protocols outlined in this section were approved by the local ethical review committee of the University of Oxford’s Department of Zoology.

#### Subjects

Twenty homing pigeons aged 1 or 3 years were used. Body size was quantified by measuring tarsus length (mm) and body mass (g). Tarsus length was measured with callipers sensitive to 0.1 mm using the methods described by Sutherland and colleagues [22]. Body mass was measured using digital scales (± 1 g; Salter ARC Electronic Kitchen Scales, Salter, UK). All subjects completed a minimum of 15 solo flights from the release site used in this study immediately preceding the start of the experiment. The subjects were housed with approximately 120 other pigeons in 2 neighbouring lofts at the Oxford University Field Station, Wytham, UK (51°46’58.2” N, 1°19’2.7” W). Access to water, grit, and a standard pigeon feed mix were available ad libitum at all times in the loft.

#### Data logging

The birds were tracked using 5 Hz GPS loggers (15 g; QStarz BT-Q1300ST, Qstarz International, Taipei, Taiwan) and 200 Hz tri-axial accelerometers (± 16*g*; 11 g; Axivity AX3, Axivity, Newcastle upon Tyne, UK), which were attached via Velcro strips glued to trimmed feathers on the birds’ backs. In total, the loggers and fastenings weighed 27 g. To enable subjects to adapt to carrying the additional mass, clay weights were attached to them throughout the pretraining and experimental periods, which meant the weights were attached for a minimum of 43 days prior to the start of the experiment. The weights were exchanged for the loggers immediately prior to each release. The GPS units have an approximately 3 m accuracy, which means that 50% of position fixes will fall within 3 m of the true position within the world coordinate system [39]. GPS and accelerometer data were downloaded using QTravel (version 1.48(T); Qstarz International, Taipei, Taiwan) and Open Movement (Om) GUI Application (version 1.0.0.28; Axivity, Newcastle upon Tyne, UK), respectively.

The weather, including mean wind speed per minute (ms^−1^), a running mean of the wind bearing over the previous 10 minutes, temperature (^o^C), humidity (%), and barometric pressure (hPa), were recorded using a WS2083 Professional Wireless Weather Station with USB upload (Aercus Instruments, Auckland, New Zealand) situated 5.5 m above the ground near the pigeon lofts and Cumulus Weather Station Software (version 1.9.4; Sandaysoft, Sanday, Orkney, UK).

#### Experimental procedures

The release site was located 7.06 km from the loft on a bearing of 282° (Barnard Gate; 51°47’48.1” N, 1°25’3.3” W). The experiment consisted of 4 phases: Phase 1: 6 individual releases (solo 1); Phase 2: 6 releases with a bird of a similar size (similar-sized pair); Phase 3: 6 releases with a bird of a different size (different-sized pair); and Phase 4: 6 individual releases (solo 2). Bird pairings can be found in S3 Table. Releases were conducted between June and September 2015, on days when the sun was visible and the wind speed was <7 ms^−1^. Subjects participated in a maximum of 2 releases per day, with a minimum of 3 hours between each release. The birds had to complete a minimum of 1 minute flying together for the flight to be included in the analysis. If the birds spent less than 1 minute together, the flight was repeated. In total, 6 releases (5%) out of 116 pair releases in which birds did not separate at the start had to be repeated. One different-sized pair did not complete the final pair release after repeatedly separating. The Velcro failed on bird S27 after the third release with a different-sized bird; therefore the pairing S27 and S84 only completed 3 different size pair releases and S27 did not complete the final solos. In addition, S13 only completed one final solo before the Velcro failed, S87 completed 4 final solos, and S05 and S25 completed 5 final solos each. The remaining 15 birds all completed the final solo releases.

#### Data processing

Data were processed using the procedures outlined in Taylor and colleagues [21]. For each GPS point, the orthodromic (great-circular) distance travelled and birds’ final bearing from the previous point were calculated using the haversine formula and forward azimuth, respectively. The dorsal accelerometer measurements were filtered by taking a running mean over 3 data points (0.015 s). Static acceleration (or gravity) was removed by subtracting a running mean over 15 wingbeat cycles (>2 s). The wingbeat frequency (number of wingbeats per second; Hz) and peak-to-peak dorsal body acceleration (*g*) using the dorsal acceleration signal (Z-axis) were calculated for each individual wingbeat. The amplitude of the dorsal body displacement (mm) was then calculated by the double integration of dorsal accelerometer measurements [14,21]. In addition, we calculated the duration of the downstroke from the peak downstroke force (maximum *g*-force) to the lower reversal point (minimum *g*-force). The upstroke phase duration, which included the start of the downstroke, was measured from minimum *g*-force to the maximum. We used the maximum and minimum *g*-force peaks to divide the wingbeat for consistency, because the start of the kinematic downstroke was not distinguishable in the data from paired flights. See S1 Text for further analysis.

Wind support, crosswind, and airspeed were calculated using the methods described in Safi and colleagues [40] using the measurements from the weather station and speed derived from the GPS devices. Humid air density (kg m^−3^; *ρ*_*air*_) was calculated from measures of barometric pressure (hPa; *P*), temperature (^o^C; *T*_*C*_), and relative humidity (%; ϕ) recorded by the weather station, using the following calculation derived from the ideal gas law:

$$\rho_{air}=\left(\frac{P_{d}}{R_{d}T}\right)+\left(\frac{P_{v}}{R_{v}T}\right),$$

where *P*_*d*_ is pressure of dry air (Pa), *R*_*d*_ is gas constant for dry air (287.05 J kg^−1^ K^−1^), *P*_*v*_ is pressure of water vapour (Pa), *R*_*v*_ is the gas constant for water vapour (461.495 J kg^−1^ K^−1^), and *T* is ambient temperature (K). *P*_*v*_ can be calculated from the saturation of vapour pressure (*P*_*sat*_) and relative humidity (ϕ):

$$P_{v}=\phi P_{sat}.$$

We used the Arden-Buck [41,42] equation to calculate *P*_*sat*_, where *P*_*sat*_ (hPa) is calculated as:

$$P_{sat}=0.61121\;\exp\left(\left(18.678-\frac{T_{C}}{234.5}\right)(\frac{T_{C}}{257.14+T_{C}})\right).$$

*P*_*d*_ can then be calculated from the barometric pressure (*P*) and the vapour pressure of water (*P*_*v*_):

$$P_{d}=P-P_{v}.$$

The birds’ route accuracy was calculated using a weighted mean cosine of the angle (*θ*) between the birds bearing and the bearing to the loft for each timestep, where *θ* is equal to the smallest angle difference so that *θ* ranged from 0 (heading directly to the loft) and 180 (heading directly away from the loft), and the orthodromic distance between each GPS point (*d*) using the following calculation:

$$\frac{1}{D}\sum_{i=1}^{n}d_{i}\cos\theta_{i},$$

where *D* is the total distance flown. Route accuracy is, therefore, on a scale of −1 (heading in a straight line away from the destination) to 1 (straight line to the destination). For orientated movement, route accuracy is >0, which means route accuracy is akin to the straight-line index [43] but enables us to calculate the accuracy for sections of a flight, rather than a whole flight, which is necessary if the birds separate during the flight and fly solo.

The data were trimmed within a 200-m radius around the release site and the loft to remove take-off and landing. When analysing the pair tracks, sections of flight where the birds were ≥50 m apart were excluded. In addition, if the birds swapped front-versus-back positions, the bird who spent the majority of the flight in front based on GPS positioning was identified and the rest of the data from when the other bird was in front was excluded.

#### Data analysis

We analysed the data using Bayesian hierarchical models, which are analogous to mixed models in frequentist methods and enabled us to account for the effects of each bird both as an individual and a partner in a specific pair. The median wingbeat frequency (${\bar{W}}_{i,j}$) for the pair (*i*,*j*) was assumed to be normally distributed,

$${\bar{W}}_{i,j}∼N(\delta_{0}1_{pair}+\delta_{1}\|T_{i}-T_{j}\|+\omega_{i}\mu_{i}+(1-\omega_{i})\mu_{j}+\gamma X,\sigma ),$$

where 1_*pair*_ is an indicator variable equal to 1 if the bird flew in a pair, and 0 for solo flights. *δ*_0_ is the difference in wingbeat frequency between the solo and paired flight. *δ*_1_ is the difference in wingbeat frequency for every mm absolute difference in tarsus length (*T*) between the pair (*i*,*j*). For solo flights, the term involving *δ*_1_ equals zero. The expression *ω*_*i*_*μ*_*i*_+(1−*ω*_*i*_)*μ*_*j*_ represents a weighted average of the solo wingbeat frequency (*μ*) of birds *i* and *j* with a mixing weighting (*ω*_*i*_), which determines the weight placed on the bird’s own solo wingbeat frequency (*μ*_*i*_) relative to that of its partner (*μ*_*j*_). For solo flights, this weighted average equals bird *i*’s solo wingbeat frequency (*μ*_*i*_). The weighting is bounded to lie between 0 and 1 and was determined by a logistic sigmoid function of the absolute difference in tarsus length,

$$\omega_{i}={logit}^{-1}(\eta_{1}+\eta \|T_{i}-T_{j}\|).$$

However, across all cases, there was no consistent effect of tarsus difference on the mixing weighting.

Finally, *γX* represents the effect of the covariates, which accounts for median wind support (ms^−1^), median crosswind (ms^−1^), median temperature (^o^C), median humidity (%), humid air density (kg m^−3^), and the date of release treated as a categorical variable. The birds’ median airspeed (ms^−1^) was also added as a covariate on all models except for models of airspeed. We used airspeed rather than ground speed as a covariate because ground speed and wind support were correlated (S5 Fig). In terms of the response variable, there was almost no difference between the models of airspeed and ground speed as the model accounts for the effect of wind (0.63, 95% credible interval [0.07, 1.19] compared to 0.70, 95% credible interval [0.10, 1.28]). For consistency with the covariates, we present the results of the model for airspeed. A covariate indicating whether the bird was in front or behind was also added to the model in a secondary analysis to determine the effect of the birds’ position on wingbeat frequency.

In addition to modelling the median values, we also took a random sample of 100 individual wingbeats to analyse the effect of horizontal distance between birds in pairs. We analysed horizontal distance rather than 3-dimensional distance because GPS precision is generally poorer in the vertical than the horizontal [44]. Horizontal distance (m) was added as a covariate to the paired data, along with a categorical covariate identifying the specific bird and flight to account for the repeated measures of 50 wingbeats from 1 flight. In total, 44,500 wingbeats from 454 unique bird and flight combinations were analysed.

To investigate whether the birds were flying in phase, we used the following model to identify whether the median wingbeat frequency of the bird behind (${\bar{W}}_{B}$) is related to the bird in front (${\bar{W}}_{F}$) in pair (*P*):

$${\bar{W}}_{B}∼N(\alpha_{P}+\beta_{P}{\bar{W}}_{F}+\gamma X,\sigma^{\prime }).$$

We also investigated whether the difference between the bird in front and the bird behind’s tarsus length, body mass, or solo airspeed (*S*) individually predicted why the bird was in front (*F*) in the pair using a Bernoulli regression:

$$F_{i}∼{Bernoulli(\alpha }_{P}+\beta_{P}S_{i}).$$

The model priors were centred on the null hypothesis using the mean, SD, and square root SD of the solo data (S4 Table). Eight Markov chain Monte Carlo (MCMC) chains were run simultaneously, each with 12,500 warm-up and 12,500 model iterations, which resulted in 100,000 samples for each posterior distribution. For the model involving raw wingbeat data, the model was run for 10,000 samples due to the size of the model. Across all estimated models and parameters, we detected convergence in the sampling distribution as determined by using a criterion $\hat{R}\leq 1.1$ on all parameters [45]. The number of divergent iterations was 0.0% to 1.3% of the total sample size. The code for the wingbeat frequency model and the model output can be found in the S2 Text.

In addition to these results, 1 similar-sized pair (birds B01 and B82 with a tarsus length of 32.4 mm and 33.3 mm, respectively) and 1 different-sized pair (birds B01 and B07 with a tarsus length of 32.4 mm and 35.2 mm, respectively) split and flew solo for more than 30% of the flight for 3 of their 6 releases. As the sample size is low, only descriptive statistics can be performed comparing the paired (<50 m distance between birds) and solo flight (>300 m distance).

We approximated the number of wingbeats difference between the solo and paired flight using the total flight distance (excluding the 200-m take-off and landing) and the model results from route accuracy, airspeed, and wingbeat frequency (S2 Table). We calculated the number of wingbeats difference because not all of birds completed 100% of the paired flights together or with one leader.

Data processing and analysis were conducted using MATLAB (version R2015a; MathWorks, Natick, USA) and the open-source software R (version 3.4.2) [46]. Bayesian models were written in Stan [47] using the R interface RStan (version 2.16.2) [48].

#### Calculation of aerodynamic power requirements

We estimated the aerodynamic power requirements of our birds using 2 established models from the bird flight literature. We first implemented Pennycuick’s classical theory [26], using the current version of the accompanying software Flight 1.25 for Windows. We then implemented a new optimal vortex wake theory [27], using its accompanying ‘R’ package ‘afpt’ [49]. In each case, we assumed a wing span of 0.67 m and an aspect ratio of 6.9, together with the values of wingbeat frequency and/or airspeed seen in paired versus solo flight, at the default parameter settings for each program.

### Experiment 2

#### Ethics statement

The protocols outlined in this section were approved by the University of Oxford's Zoology Animal Welfare Ethical Review Board (No. APA/1/5/ZOO/NASPA/Biro/ PigeonsHeadmountedsensors).

#### Subjects

Six homing pigeons aged 3 to 10 years old were used. The pigeons were held under the same conditions as outlined above and all had experience of experimental releases and the release site.

#### Data logging

We used a custom-built ‘p-Sensor’ to simultaneously record head movement and position. The p-Sensor included an IMU with a combination of a tri-axial gyroscope, tri-axial accelerometer and tri-axial magnetometer recording at 60 Hz, and a GPS logger recording at 10 Hz. The IMU was mounted using double-sided tape onto a custom-made and custom-fitted wire mask designed to fit each bird’s head. The GPS logger, SD card, battery, and microcomputer were placed in an elasticated backpack on the birds back. The instrumentation, mask, and backpack weighed 28.1 g and constituted 4.9% of the body mass of the smallest bird, of which the IMU unit on the bird’s head only weighed 1 g. For more details, see Kano and colleagues [34].

All birds were habituated to wearing the custom-made mask for at least 7 days prior to testing. For each day of habituation, the bird was fitted with a mask and carefully monitored for 2 hours within its home loft for signs of discomfort and abnormal patterns of locomotion. After 7 days of habitation in the loft, the pigeons were released outside the loft and allowed to fly freely under close observation.

#### Experimental procedures

The release site was located 0.95 km from the loft on a bearing of 199° (Wytham Woods; 51°46’29.4” N, 1°19’18.7” W). The experiments were conducted on the 23rd July 2017. Releases were only conducted when the wind was low (<5 ms^−1^) and the sun’s disc was visible. For the day of testing, the birds were fitted with a mask and allowed to habituate to wearing the mask in the home loft before being transported to the release site by car. The birds were released once solo and once in a pair on the same day. The release order was randomised.

#### Data processing and analysis

The data processing was conducted as outlined in Taylor and colleagues [21]. Vertical (Z-axis) accelerometer measurements were smoothed by taking a running mean over 5 data points (0.083 s) and then filtered using a 4th order high-pass Butterworth filter with a cut-off frequency of 1 Hz. The peak-to-peak vertical head displacement was calculated by the double integration of the vertical accelerometer measurements. We compared the median peak-to-peak vertical head displacement between solo and paired releases for each bird.

The data underlying the statistical analyses can be found in S1 Data, and the raw data are deposited in the Dryad Digital Repository: https://doi.org/10.5061/dryad.m7253n8 [23].

## Supporting information

S1 Fig Bayesian hierarchical model results. Graphs show the difference (Δ) in (A) wingbeat frequency, (B) peak-to-peak DB acceleration, (C) downstroke phase duration, (D) upstroke phase duration, (E) DB amplitude, and (F) airspeed between solo and paired flight per mm difference in tarsus length. Black line corresponds to the mean model fit, which represents the difference between solo and paired flight per mm difference in tarsus length. Grey shaded area corresponds to the 95% credible interval of both the difference between solo and paired flight and the difference per mm difference in tarsus length. Points correspond to the difference between the median value for the paired flight and the median value for the solo flight, after accounting for the effects of weather, day, and, in case of (A–C) airspeed. Dotted line shows the solo value. All axes scaled to show ±40% of mean solo value. Data deposited in S1 Data and model code deposited in S2 Text. DB, dorsal body.
(EPS)

S2 Fig **Probability of a bird leading per unit difference in (A) tarsus length, (B) median solo airspeed, and (C) body mass.** Data deposited in S1 Data. See Materials and methods for model description.
(EPS)

S3 Fig Median wingbeat frequency of the bird in front and bird behind during each flight. The different coloured lines correspond to least squares regression lines fitted to the data from each pair, and the lack of any direct relationship indicates that the bird’s wingbeats cannot be phase-locked through the flight. Data deposited in S1 Data.
(EPS)

S4 Fig **Raw data showing the results per release for the Bayesian hierarchical model covariates: (A) median airspeed (ms**^**−1**^**), (B) median temperature (**^**o**^**C), (C) median wind support (ms**^**−1**^**), (D) median crosswind, (E) median humidity (%), and (F) median humid air density (kg m**^**−3**^**).** Data deposited in S1 Data.
(EPS)

S5 Fig Relationship between ground speed, airspeed, wind support, and crosswind, with bivariate scatter plots below the diagonal, histograms on the diagonal, and the Pearson correlation above the diagonal. Data deposited in S1 Data.
(EPS)

S1 Table Results of the Bayesian hierarchical models. Corrected solo mean values refer to the mean of the median value from each individual, which has been corrected for the effects of airspeed, wind support, crosswind, temperature, humidity, and air density according to the results of the model. Note: the covariates in this table are the difference per unit difference in the covariate. Data deposited in S1 Data and model code deposited in S2 Text.
(XLSX)

S2 Table A calculation of the total number of wingbeats that would have been used during solo and paired flight had the birds flown with the stated wingbeat frequency, mean route accuracy, and at the stated mean airspeed in the absence of any wind support. We calculated the difference using the results of the Bayesian hierarchical models combined with the means of the solo flights after accounting for the effects of covariates as not all birds completed 100% of the paired flights together with a single leader. Data deposited in S1 Data.
(XLSX)

S3 Table Bird and pairing information, including tarsus length (mm), body mass (g), and median solo airspeed (ms^−1^). (XLSX)

S4 Table Model priors for the hyperparameters of the Bayesian hierarchical model. The width of the prior distributions was chosen to be similar to that of the observed quantities. The hyperparameters represent the population (i.e., across all birds) averages. The bird-level parameters are obtained by sampling from a normal distribution conditional on the hyperparameters. See S2 Text for the model code and Materials and methods for further details.
(XLSX)

S1 Text Analysis of accelerometer output. (DOCX)

S2 Text **(A) Stan code and (B) example model output for the Bayesian hierarchical model of median wingbeat frequency.** Data available in S1 Data.
(DOCX)

S1 Data Data underlying the statistical analyses from Experiment 1. (XLSX)

## Abbreviations

GPS: global positioning system
MCMC: Markov chain Monte Carlo
