# Supplementary material for: Birds invest wingbeats to keep a steady head and reap the ultimate benefits of flying together
Source: PLoS Biol. 2019 Jun 18;17(6):e3000299. doi: 10.1371/journal.pbio.3000299 (PMC6581236; doi:10.1371/journal.pbio.3000299)
Supplement: S1 Text — (DOCX) [file pbio.3000299.s010.docx]

**Supplementary Information**

**Birds invest wingbeats to keep a steady head and reap the ultimate benefits of flying together**

Lucy A. Taylor, Graham K. Taylor, Ben Lambert, James A. Walker, Dora Biro

and Steven J. Portugal

**S1 Text.** Analysis of accelerometer output

The purpose of this section is to provide a rigorous analysis of the oscillatory accelerations experienced by an accelerometer worn on a bird’s back during flapping flight. Here, the axis of the accelerometer is assumed to be aligned dorsoventrally on the bird, and possible angular changes in the inclination of this axis with respect to the Earth are assumed to be sufficiently small or slow that the dorsoventral component of gravitational acceleration that the accelerometer experiences can be considered constant and hence neglected in an analysis of the oscillatory accelerations.

Under Newton’s Second Law, an accelerometer placed at the centre of mass of an object experiences an acceleration $a\left( t \right)$ equal to the net external force on the object divided by its mass. If the accelerometer is placed away from the centre of mass, then the angular motion of the bird will also contribute to the sensed acceleration, but this effect may be neglected in non-manoeuvring flight. Under sinusoidal aerodynamic forcing, the accelerometer therefore experiences an aerodynamic acceleration:

$$\tilde{a}_{A}\left( t \right)=\frac{F\sin\left( 2\pi ft \right)}{m}$$

(Eq. S1)

where $F$ is the aerodynamic forcing amplitude, $f$ is the wingbeat frequency, $t$ is time, and $m$ is the mass. Integrating twice with respect to time, this aerodynamic acceleration is associated with an oscillatory displacement of the centre of mass:

$$\tilde{d}_{A}\left( t \right)=-\frac{F\sin\left( 2\pi ft \right)}{4\pi^{2}f^{2}m}$$

(Eq. S2)

Other things being equal, the aerodynamic displacement of the centre of mass therefore scales as $f^{-2}$, such that the 18% increase in wingbeat frequency that we measured between solo and paired flights (Table S1) could be expected to cause a 28% reduction in the amplitude of the aerodynamic body displacement. This conclusion holds provided that the increase in wingbeat frequency is not accompanied by any increase in the amplitude $F$ of the dorsoventral aerodynamic forcing, which is a reasonable assumption in sustained level flight, for which the time-averaged vertical force must always balance the weight of the bird. This being so, it follows that a bird can reduce the bobbing motion that its head or body experiences in flight simply by increasing its wingbeat frequency.

A bird is not a rigid body, however, and flapping its wings will cause its overall mass distribution to vary periodically. An accelerometer placed at any anatomically fixed point on the bird’s body will therefore experience an additional inertial acceleration due to the displacement of the bird’s body in relation to its overall centre of mass. The wings of a pigeon together comprise 13% of its overall mass, and their respective centres of mass move on radii of 0.07m from the shoulder joint [1]. Lowering the wings from the extreme dorsal to the extreme ventral position will therefore raise the bird’s body by 0.018m relative to its overall centre of mass, calculated as 13% of the peak-to-peak displacement of the wing’s centre of mass. Based on the wingtip kinematics presented by Tobalske & Dial [2], an inertial displacement nearer 80% of this extreme seems realistic at the 20ms^-1^ airspeed that we observed (Table S1), bringing the expected peak-to-peak amplitude of the oscillatory inertial displacement $\tilde{d}_{I}\left( t \right)$ to approximately 0.015m. Since the amplitude of this displacement is independent of the wingbeat frequency, the presence of this inertial forcing is expected to attenuate the overall reduction of body displacement that would otherwise result from increasing the frequency of the aerodynamic forcing, which qualitatively speaking is what we observed (see below).

Neither the aerodynamic nor the inertial component of the total displacement is expected to be perfectly sinusoidal, but it is nevertheless informative to consider how their waveforms would be expected to combine if as a good first approximation they were. Approximating the dorsoventral motion of the wing’s centre of mass as a simple sinusoid yields the following expression for the inertial displacement:

$$\tilde{d}_{I}\left( t \right)\approx0.0075\sin\left( 2\pi ft+\varphi\right)$$

(Eq. S3)

where $\varphi$ is the phase of the motion with respect to the aerodynamic displacement $\tilde{d}_{A}\left( t \right)$ in Eq. S2, and where the numerical constant is half the peak-to-peak amplitude. Because the upward aerodynamic acceleration $\tilde{a}_{A}\left( t \right)$ of the accelerometer is expected to peak at mid-downstroke, its upward aerodynamic displacement $\tilde{d}_{A}\left( t \right)$ is expected to peak at mid-upstroke (see Eq. S2). Hence, since the upward inertial displacement of the accelerometer $\tilde{d}_{I}\left( t \right)$ peaks at the bottom of the downstroke, it follows that the inertial displacement $\tilde{d}_{I}\left( t \right)$ leads the aerodynamic displacement $\tilde{d}_{A}\left( t \right)$ by approximately 90˚ such that $\varphi=-\pi/2$.

The amplitude of a waveform combining two sinusoids with a 90˚ phase lag is just the square root of the sum of the squared amplitudes of the two sinusoids. Subtracting the squared peak-to-peak amplitude of the predicted displacement due to inertial forcing (0.015 m) from the squared peak-to-peak amplitude of the measured displacement (0.027 m in solo flight *versus* 0.022 m in paired flight; Table S1) and taking the square root, we therefore arrive at a robust empirical estimate of the peak-to-peak amplitude of the displacement due to aerodynamic forcing, which is 0.022m in solo flight and 0.016m in paired flight. The resulting empirical estimate of a 27% reduction in the peak-to-peak amplitude of the aerodynamic displacement between solo and paired flight coincides almost exactly with the 28% reduction that we predicted theoretically from the accompanying 18% increase in wingbeat frequency using Eq. S2 above. These two estimates are mathematically independent, and therefore provide a useful internal validation of our analysis. Moreover, these figures are modified only slightly if we assume that flying in pairs is associated with a 10% decrease in the wingbeat amplitude, as is argued in the main text. Multiplying our empirical estimates of the peak-to-peak amplitude of the aerodynamic displacement by the factor $4\pi^{2}f^{2}$ in the denominator of Eq. S2, we arrive at an estimate of 2.7*g* for the peak-to-peak amplitude of the acceleration due to aerodynamic forcing in solo flight, and 2.8*g* in paired flight. This is consistent with our earlier argument that the amplitude $F$ of the dorsoventral aerodynamic forcing should have been similar in paired and solo flight, on the basis that the time-averaged vertical aerodynamic force must have balanced the weight of the bird under both flight conditions (see also the main text).

1. van den Berg C, Rayner J. The moment of inertia of bird wings and the inertial power requirement for flapping flight. J Exp Biol. 1995;198: 1655–64. Available: http://www.ncbi.nlm.nih.gov/pubmed/9319563

2. Tobalske BW, Dial KP. Flight kinematics of black-billed magpies and pigeons over a wide range of speeds. J Exp Biol. 1996;199: 263–280. Available: http://www.ncbi.nlm.nih.gov/pubmed/9317775
