# Supplementary material for: Birds invest wingbeats to keep a steady head and reap the ultimate benefits of flying together
Source: PLoS Biol. 2019 Jun 18;17(6):e3000299. doi: 10.1371/journal.pbio.3000299 (PMC6581236; doi:10.1371/journal.pbio.3000299)
Supplement: S2 Text — (A) Stan code and (B) example model output for the Bayesian hierarchical model of median wingbeat frequency. Data available in S1 Data. (DOCX) [file pbio.3000299.s011.docx]

**Supplementary Information**

**Birds invest wingbeats to keep a steady head and reap the ultimate benefits of flying together**

Lucy A. Taylor, Graham K. Taylor, Ben Lambert, James A. Walker, Dora Biro

and Steven J. Portugal

**S2 Text.** (a) Stan code and (b) example model output for the Bayesian hierarchical model

1. Stan code for the Bayesian hierarchical model of wingbeat frequency.

In the model, the bird-level parameters are sampled from a normal distribution conditional on the hyper-parameters, which represent the population averages. For example, delta_i_ ~ N(delta^{top}^, sigma_delta^{top}^), where delta_i_ is the uplift for bird $i$of being in a pair, and delta^{top}^ and sigma_delta^{top}^ represent the population mean uplift and the standard deviation of the individual birds' effects about this mean. Note that in the Stan model, we use a non-centred parameterisation to implement this model, which helps with sampling.

functions{

// calculates mixing weight of solo frequencies of birds

// based on difference in tarsus length

real weighting(int bird_a, int bird_b, real[] chi_0, real[] chi, real[] tarsus){

real omega;

omega = inv_logit(chi_0[bird_a] + chi[bird_a] * fabs(tarsus[bird_a] - tarsus[bird_b]));

return(omega);

}

}

data{

int N; // length of data

real W[N]; // wingbeat frequency

int nBirds; // number of birds

// indices

int bird1[N]; // identity of first bird in pair

int bird2[N]; // identity of second bird in pair -- can be same as first

// covariates

real wind1[N];

real wind2[N];

real temp[N];

real density[N];

real humidity[N];

real airspeed[N];

// matrix covariates

int<lower=1> K;

matrix[N, K] date;

// size covariates

real tarsus[nBirds];

}

parameters{

// bird-specific

real mu[nBirds]; // mean wingbeat for each bird

real delta_0_raw[nBirds]; // uplift from being in a pair

real eta_raw[nBirds]; // effect of size difference on wingbeat

// top-level

real delta_0_top;

real eta_top;

real<lower=0> sigma_delta_0_top;

real<lower=0> sigma_eta_top;

// covariate specific

real gamma1;

real gamma2;

real gamma3;

real gamma4;

real gamma5;

real gamma6;

// matrix covariate

vector[K] beta;

// other

real<lower=0> sigma;

// Leading parameter

real chi_raw[nBirds];

real chi_0_raw[nBirds];

real chi_top;

real chi_0_top;

real<lower=0> chi_sigma_top;

real<lower=0> chi_0_sigma_top;

}

transformed parameters{

real chi[nBirds];

real chi_0[nBirds];

real delta_0[nBirds]; // uplift from being in a pair

real eta[nBirds]; // effect of size difference on wingbeat

for(i in 1:nBirds){

chi_0[i] = chi_0_top + chi_0_raw[i] * chi_0_sigma_top;

chi[i] = chi_top + chi_raw[i] * chi_sigma_top;

delta_0[i] = delta_0_top + delta_0_raw[i] * sigma_delta_0_top;

eta[i] = eta_top + eta_raw[i] * sigma_eta_top;

}

}

model{

real omega;

for(i in 1:N){

real aMean;

if(bird1[i] == bird2[i]){ // solo

aMean = 0.0;

else{

aMean = delta_0[bird1[i]] + eta[bird1[i]] * fabs(tarsus[bird1[i]] - tarsus[bird2[i]]);

}

// mixing probability

omega = weighting(bird1[i], bird2[i], chi_0, chi, tarsus);

// likelihood

W[i] ~ normal(aMean + omega * mu[bird1[i]] + (1 - omega) * mu[bird2[i]]

+ wind1[i] * gamma1 + wind2[i] * gamma2 + temp[i] * gamma3

+ density[i] * gamma4 + humidity[i] * gamma5 + airspeed[i] * gamma6

+ date[i] * beta, sigma);

}

// priors

chi_0_raw ~ normal(0, 1);

chi_raw ~ normal(0, 1);

delta_0_raw ~ normal(0, 1);

eta_raw ~ normal(0, 1);

mu ~ normal(5.5, 0.3); // change mean to be similar to that observed + sd change

gamma1 ~ normal(0, 0.6);

gamma2 ~ normal(0, 0.6);

gamma3 ~ normal(0, 0.6);

gamma4 ~ normal(0, 0.6);

gamma5 ~ normal(0, 0.6);

gamma6 ~ normal(0, 0.6);

beta ~ normal(0, 0.6);

sigma ~ cauchy(0,0.6);

// hyper-priors

chi_0_top ~ normal(0, 2);

chi_top ~ normal(0, 2);

chi_sigma_top ~ cauchy(0, 2);

chi_0_sigma_top ~ cauchy(0, 2);

delta_0_top ~ normal(0, 0.3);

eta_top ~ normal(0, 0.3);

sigma_delta_0_top ~ cauchy(0, 0.6);

sigma_eta_top ~ cauchy(0, 0.6);

}

generated quantities{

real delta_0_av;

real eta_av;

real chi_av;

real chi_0_av;

delta_0_av = normal_rng(delta_0_top, sigma_delta_0_top);

eta_av = normal_rng(eta_top, sigma_eta_top);

chi_av = normal_rng(chi_top, chi_sigma_top);

chi_0_av = normal_rng(chi_0_top, chi_0_sigma_top);

}

1. Model output for median wingbeat frequency

Inference for Stan model: wingbeat_continuous_pred_MEDIAN_FPS_wDate.

8 chains, each with iter=25000; warmup=12500; thin=1;

post-warmup draws per chain=12500, total post-warmup draws=1e+05.

|  | mean | se_mean | sd | 2.5% | 25% | 50% | 75% | 97.5% | n_eff | Rhat |
| --- | --- | --- | --- | --- | --- | --- | --- | --- | --- | --- |
| mu[1] | 5.48 | 0.00 | 0.08 | 5.32 | 5.42 | 5.48 | 5.54 | 5.64 | 14334 | 1 |
| mu[2] | 5.33 | 0.00 | 0.08 | 5.16 | 5.27 | 5.33 | 5.38 | 5.49 | 14362 | 1 |
| mu[3] | 5.50 | 0.00 | 0.08 | 5.33 | 5.44 | 5.50 | 5.56 | 5.66 | 14261 | 1 |
| mu[4] | 5.69 | 0.00 | 0.08 | 5.52 | 5.63 | 5.69 | 5.74 | 5.85 | 14708 | 1 |
| mu[5] | 5.09 | 0.00 | 0.09 | 4.92 | 5.03 | 5.09 | 5.14 | 5.25 | 15027 | 1 |
| mu[6] | 5.43 | 0.00 | 0.08 | 5.27 | 5.37 | 5.43 | 5.49 | 5.60 | 14672 | 1 |
| mu[7] | 5.77 | 0.00 | 0.08 | 5.60 | 5.71 | 5.77 | 5.82 | 5.93 | 14371 | 1 |
| mu[8] | 5.18 | 0.00 | 0.08 | 5.01 | 5.12 | 5.18 | 5.23 | 5.34 | 14325 | 1 |
| mu[9] | 5.47 | 0.00 | 0.09 | 5.30 | 5.41 | 5.47 | 5.53 | 5.64 | 14640 | 1 |
| mu[10] | 5.65 | 0.00 | 0.08 | 5.49 | 5.60 | 5.65 | 5.71 | 5.82 | 14588 | 1 |
| mu[11] | 5.40 | 0.00 | 0.08 | 5.23 | 5.34 | 5.40 | 5.45 | 5.56 | 14629 | 1 |
| mu[12] | 5.37 | 0.00 | 0.09 | 5.20 | 5.31 | 5.37 | 5.43 | 5.54 | 15410 | 1 |
| mu[13] | 5.63 | 0.00 | 0.09 | 5.46 | 5.57 | 5.63 | 5.69 | 5.80 | 14966 | 1 |
| mu[14] | 5.45 | 0.00 | 0.08 | 5.29 | 5.39 | 5.45 | 5.51 | 5.61 | 14297 | 1 |
| mu[15] | 5.35 | 0.00 | 0.09 | 5.17 | 5.29 | 5.35 | 5.42 | 5.53 | 17287 | 1 |
| mu[16] | 5.65 | 0.00 | 0.09 | 5.48 | 5.59 | 5.65 | 5.71 | 5.82 | 15320 | 1 |
| mu[17] | 5.52 | 0.00 | 0.10 | 5.33 | 5.45 | 5.52 | 5.58 | 5.71 | 19080 | 1 |
| mu[18] | 5.76 | 0.00 | 0.08 | 5.60 | 5.70 | 5.76 | 5.82 | 5.92 | 14270 | 1 |
| mu[19] | 5.73 | 0.00 | 0.08 | 5.57 | 5.68 | 5.73 | 5.79 | 5.90 | 14512 | 1 |
| mu[20] | 5.48 | 0.00 | 0.09 | 5.30 | 5.42 | 5.48 | 5.54 | 5.65 | 16135 | 1 |
| delta_0_raw[1] | 0.62 | 0.00 | 0.71 | -0.79 | 0.15 | 0.62 | 1.09 | 2.00 | 40232 | 1 |
| delta_0_raw[2] | 0.29 | 0.00 | 0.57 | -0.87 | -0.07 | 0.30 | 0.66 | 1.41 | 62888 | 1 |
| delta_0_raw[3] | -0.97 | 0.00 | 0.58 | -2.15 | -1.34 | -0.96 | -0.58 | 0.14 | 64710 | 1 |
| delta_0_raw[4] | -0.53 | 0.00 | 0.55 | -1.67 | -0.88 | -0.51 | -0.17 | 0.51 | 56926 | 1 |
| delta_0_raw[5] | -0.19 | 0.00 | 0.61 | -1.48 | -0.56 | -0.16 | 0.21 | 0.95 | 50776 | 1 |
| delta_0_raw[6] | -0.09 | 0.00 | 0.55 | -1.20 | -0.45 | -0.09 | 0.27 | 0.97 | 55864 | 1 |
| delta_0_raw[7] | 0.37 | 0.00 | 0.57 | -0.76 | 0.00 | 0.38 | 0.75 | 1.48 | 58506 | 1 |
| delta_0_raw[8] | -0.46 | 0.00 | 0.69 | -1.79 | -0.92 | -0.47 | 0.00 | 0.90 | 62466 | 1 |
| delta_0_raw[9] | -1.16 | 0.00 | 0.73 | -2.51 | -1.65 | -1.20 | -0.72 | 0.41 | 50464 | 1 |
| delta_0_raw[10] | 1.48 | 0.00 | 0.58 | 0.35 | 1.10 | 1.47 | 1.86 | 2.65 | 66891 | 1 |
| delta_0_raw[11] | 0.60 | 0.00 | 0.54 | -0.48 | 0.24 | 0.59 | 0.94 | 1.67 | 58151 | 1 |
| delta_0_raw[12] | -0.50 | 0.00 | 0.53 | -1.60 | -0.83 | -0.48 | -0.14 | 0.49 | 58767 | 1 |
| delta_0_raw[13] | -0.65 | 0.00 | 0.57 | -1.81 | -1.01 | -0.63 | -0.27 | 0.45 | 46659 | 1 |
| delta_0_raw[14] | 1.22 | 0.00 | 0.53 | 0.24 | 0.87 | 1.21 | 1.56 | 2.31 | 69635 | 1 |
| delta_0_raw[15] | 0.81 | 0.00 | 0.57 | -0.31 | 0.44 | 0.81 | 1.18 | 1.94 | 58401 | 1 |
| delta_0_raw[16] | 0.50 | 0.00 | 0.57 | -0.61 | 0.13 | 0.49 | 0.87 | 1.67 | 63362 | 1 |
| delta_0_raw[17] | -0.17 | 0.00 | 0.54 | -1.28 | -0.51 | -0.16 | 0.18 | 0.87 | 60178 | 1 |
| delta_0_raw[18] | 0.72 | 0.00 | 0.53 | -0.28 | 0.37 | 0.70 | 1.06 | 1.80 | 61648 | 1 |
| delta_0_raw[19] | -1.11 | 0.00 | 0.59 | -2.31 | -1.49 | -1.10 | -0.72 | 0.03 | 63299 | 1 |
| delta_0_raw[20] | 1.11 | 0.00 | 0.71 | -0.34 | 0.66 | 1.12 | 1.57 | 2.48 | 67543 | 1 |
| eta_raw[1] | 0.68 | 0.00 | 0.86 | -1.13 | 0.15 | 0.71 | 1.25 | 2.33 | 64191 | 1 |
| eta_raw[2] | 0.15 | 0.00 | 0.81 | -1.50 | -0.36 | 0.15 | 0.68 | 1.76 | 79173 | 1 |
| eta_raw[3] | -0.21 | 0.00 | 0.77 | -1.73 | -0.69 | -0.22 | 0.26 | 1.36 | 70546 | 1 |
| eta_raw[4] | -0.08 | 0.00 | 0.97 | -1.97 | -0.73 | -0.09 | 0.56 | 1.83 | 105288 | 1 |
| eta_raw[5] | -0.43 | 0.00 | 0.93 | -2.21 | -1.05 | -0.45 | 0.18 | 1.47 | 87914 | 1 |
| eta_raw[6] | 0.32 | 0.00 | 1.01 | -1.65 | -0.36 | 0.32 | 1.00 | 2.30 | 91774 | 1 |
| eta_raw[7] | -0.22 | 0.00 | 0.94 | -2.04 | -0.86 | -0.24 | 0.40 | 1.66 | 82935 | 1 |
| eta_raw[8] | 0.10 | 0.00 | 0.98 | -1.81 | -0.56 | 0.10 | 0.77 | 2.01 | 92334 | 1 |
| eta_raw[9] | -0.55 | 0.00 | 0.93 | -2.28 | -1.19 | -0.59 | 0.04 | 1.38 | 59083 | 1 |
| eta_raw[10] | -0.12 | 0.00 | 0.99 | -2.06 | -0.80 | -0.11 | 0.57 | 1.81 | 74013 | 1 |
| eta_raw[11] | 0.23 | 0.00 | 0.99 | -1.71 | -0.43 | 0.24 | 0.90 | 2.16 | 108982 | 1 |
| eta_raw[12] | 0.14 | 0.00 | 0.92 | -1.66 | -0.47 | 0.14 | 0.75 | 1.96 | 104848 | 1 |
| eta_raw[13] | 0.17 | 0.00 | 0.99 | -1.79 | -0.50 | 0.17 | 0.84 | 2.10 | 123335 | 1 |
| eta_raw[14] | 0.16 | 0.00 | 0.95 | -1.75 | -0.47 | 0.18 | 0.82 | 1.99 | 89939 | 1 |
| eta_raw[15] | 0.02 | 0.00 | 0.97 | -1.89 | -0.63 | 0.03 | 0.68 | 1.92 | 128226 | 1 |
| eta_raw[16] | -0.48 | 0.00 | 0.90 | -2.19 | -1.08 | -0.51 | 0.09 | 1.37 | 75769 | 1 |
| eta_raw[17] | 0.23 | 0.00 | 0.91 | -1.58 | -0.37 | 0.24 | 0.85 | 2.01 | 88312 | 1 |
| eta_raw[18] | -0.01 | 0.00 | 0.96 | -1.90 | -0.65 | -0.01 | 0.63 | 1.88 | 122554 | 1 |
| eta_raw[19] | -0.29 | 0.00 | 0.95 | -2.13 | -0.94 | -0.30 | 0.34 | 1.62 | 78652 | 1 |
| eta_raw[20] | 0.20 | 0.00 | 0.82 | -1.49 | -0.32 | 0.21 | 0.73 | 1.80 | 68452 | 1 |
| delta_0_top | 1.00 | 0.00 | 0.08 | 0.85 | 0.95 | 1.00 | 1.05 | 1.15 | 38893 | 1 |
| eta_top | 0.01 | 0.00 | 0.04 | -0.06 | -0.01 | 0.01 | 0.04 | 0.09 | 45953 | 1 |
| sigma_delta_0_top | 0.17 | 0.00 | 0.04 | 0.10 | 0.14 | 0.17 | 0.20 | 0.27 | 29181 | 1 |
| sigma_eta_top | 0.05 | 0.00 | 0.03 | 0.00 | 0.02 | 0.04 | 0.07 | 0.13 | 19827 | 1 |
| gamma1 | 0.02 | 0.00 | 0.02 | -0.01 | 0.01 | 0.02 | 0.03 | 0.05 | 35008 | 1 |
| gamma2 | 0.03 | 0.00 | 0.02 | -0.02 | 0.01 | 0.03 | 0.04 | 0.07 | 81875 | 1 |
| gamma3 | 0.01 | 0.00 | 0.02 | -0.02 | 0.00 | 0.01 | 0.02 | 0.04 | 22864 | 1 |
| gamma4 | -0.41 | 0.00 | 0.39 | -1.17 | -0.67 | -0.41 | -0.15 | 0.35 | 27587 | 1 |
| gamma5 | 0.00 | 0.00 | 0.00 | -0.01 | 0.00 | 0.00 | 0.00 | 0.01 | 25356 | 1 |
| gamma6 | 0.01 | 0.00 | 0.01 | -0.01 | 0.00 | 0.01 | 0.02 | 0.03 | 36467 | 1 |
| beta[1] | -0.18 | 0.00 | 0.22 | -0.61 | -0.33 | -0.18 | -0.03 | 0.25 | 26293 | 1 |
| beta[2] | -0.07 | 0.00 | 0.17 | -0.41 | -0.19 | -0.07 | 0.05 | 0.27 | 16248 | 1 |
| beta[3] | -0.04 | 0.00 | 0.16 | -0.36 | -0.15 | -0.04 | 0.07 | 0.28 | 14680 | 1 |
| beta[4] | -0.11 | 0.00 | 0.15 | -0.40 | -0.21 | -0.11 | -0.01 | 0.18 | 11277 | 1 |
| beta[5] | -0.37 | 0.00 | 0.14 | -0.64 | -0.46 | -0.37 | -0.27 | -0.09 | 10848 | 1 |
| beta[6] | -0.29 | 0.00 | 0.15 | -0.58 | -0.39 | -0.29 | -0.20 | -0.01 | 10904 | 1 |
| beta[7] | -0.40 | 0.00 | 0.16 | -0.72 | -0.51 | -0.40 | -0.29 | -0.08 | 13502 | 1 |
| beta[8] | -0.15 | 0.00 | 0.15 | -0.45 | -0.25 | -0.15 | -0.04 | 0.15 | 11885 | 1 |
| beta[9] | 0.06 | 0.00 | 0.15 | -0.23 | -0.04 | 0.06 | 0.16 | 0.35 | 10745 | 1 |
| beta[10] | 0.17 | 0.00 | 0.18 | -0.20 | 0.04 | 0.17 | 0.29 | 0.52 | 11466 | 1 |
| beta[11] | 0.11 | 0.00 | 0.14 | -0.17 | 0.01 | 0.11 | 0.20 | 0.38 | 8892 | 1 |
| beta[12] | -0.17 | 0.00 | 0.17 | -0.50 | -0.29 | -0.17 | -0.06 | 0.15 | 10778 | 1 |
| beta[13] | -0.14 | 0.00 | 0.13 | -0.41 | -0.23 | -0.14 | -0.05 | 0.12 | 9348 | 1 |
| beta[14] | -0.16 | 0.00 | 0.13 | -0.40 | -0.24 | -0.15 | -0.07 | 0.09 | 8436 | 1 |
| beta[15] | -0.07 | 0.00 | 0.14 | -0.34 | -0.17 | -0.07 | 0.02 | 0.19 | 9420 | 1 |
| beta[16] | -0.07 | 0.00 | 0.13 | -0.33 | -0.16 | -0.07 | 0.02 | 0.19 | 8706 | 1 |
| beta[17] | -0.02 | 0.00 | 0.15 | -0.31 | -0.12 | -0.02 | 0.08 | 0.27 | 10325 | 1 |
| beta[18] | 0.13 | 0.00 | 0.13 | -0.13 | 0.04 | 0.13 | 0.21 | 0.38 | 9265 | 1 |
| beta[19] | 0.01 | 0.00 | 0.19 | -0.36 | -0.12 | 0.01 | 0.14 | 0.37 | 17427 | 1 |
| beta[20] | 0.21 | 0.00 | 0.13 | -0.04 | 0.13 | 0.21 | 0.30 | 0.46 | 8675 | 1 |
| beta[21] | 0.13 | 0.00 | 0.14 | -0.14 | 0.04 | 0.13 | 0.22 | 0.39 | 8761 | 1 |
| beta[22] | 0.29 | 0.00 | 0.13 | 0.03 | 0.20 | 0.29 | 0.38 | 0.55 | 9000 | 1 |
| beta[23] | 0.05 | 0.00 | 0.17 | -0.28 | -0.07 | 0.05 | 0.16 | 0.38 | 10893 | 1 |
| beta[24] | -0.06 | 0.00 | 0.13 | -0.31 | -0.14 | -0.06 | 0.03 | 0.20 | 8823 | 1 |
| beta[25] | -0.07 | 0.00 | 0.13 | -0.33 | -0.16 | -0.07 | 0.01 | 0.18 | 8592 | 1 |
| beta[26] | 0.14 | 0.00 | 0.12 | -0.10 | 0.06 | 0.14 | 0.23 | 0.38 | 8385 | 1 |
| beta[27] | 0.11 | 0.00 | 0.12 | -0.12 | 0.03 | 0.11 | 0.19 | 0.35 | 7918 | 1 |
| beta[28] | 0.24 | 0.00 | 0.12 | -0.01 | 0.15 | 0.24 | 0.32 | 0.48 | 8162 | 1 |
| beta[29] | 0.44 | 0.00 | 0.14 | 0.17 | 0.34 | 0.44 | 0.53 | 0.70 | 9512 | 1 |
| sigma | 0.18 | 0.00 | 0.01 | 0.17 | 0.18 | 0.18 | 0.19 | 0.20 | 86363 | 1 |
| chi_raw[1] | 0.23 | 0.00 | 0.99 | -1.80 | -0.41 | 0.28 | 0.90 | 2.10 | 63261 | 1 |
| chi_raw[2] | -0.03 | 0.00 | 0.99 | -1.94 | -0.69 | -0.04 | 0.63 | 1.93 | 92371 | 1 |
| chi_raw[3] | 0.18 | 0.00 | 0.95 | -1.74 | -0.45 | 0.19 | 0.81 | 2.05 | 97067 | 1 |
| chi_raw[4] | -0.26 | 0.00 | 0.95 | -2.08 | -0.89 | -0.30 | 0.35 | 1.71 | 83840 | 1 |
| chi_raw[5] | 0.53 | 0.00 | 0.86 | -1.28 | 0.00 | 0.53 | 1.08 | 2.23 | 71157 | 1 |
| chi_raw[6] | -0.81 | 0.00 | 0.96 | -2.52 | -1.44 | -0.88 | -0.25 | 1.32 | 61600 | 1 |
| chi_raw[7] | -0.32 | 0.00 | 0.93 | -2.08 | -0.93 | -0.36 | 0.25 | 1.65 | 60304 | 1 |
| chi_raw[8] | -0.48 | 0.00 | 0.81 | -2.04 | -0.97 | -0.50 | -0.04 | 1.32 | 54548 | 1 |
| chi_raw[9] | 0.70 | 0.00 | 0.85 | -1.13 | 0.20 | 0.71 | 1.24 | 2.35 | 68655 | 1 |
| chi_raw[10] | -0.54 | 0.00 | 0.80 | -2.07 | -1.04 | -0.57 | -0.11 | 1.27 | 44800 | 1 |
| chi_raw[11] | -0.29 | 0.00 | 1.01 | -2.19 | -0.98 | -0.32 | 0.39 | 1.76 | 102486 | 1 |
| chi_raw[12] | 0.45 | 0.00 | 0.87 | -1.33 | -0.09 | 0.44 | 1.01 | 2.18 | 81877 | 1 |
| chi_raw[13] | 0.24 | 0.00 | 0.95 | -1.68 | -0.38 | 0.25 | 0.86 | 2.08 | 102775 | 1 |
| chi_raw[14] | 0.04 | 0.00 | 0.93 | -1.79 | -0.56 | 0.02 | 0.64 | 1.92 | 76011 | 1 |
| chi_raw[15] | 0.15 | 0.00 | 0.92 | -1.66 | -0.45 | 0.14 | 0.75 | 2.02 | 99915 | 1 |
| chi_raw[16] | -0.19 | 0.00 | 0.92 | -1.93 | -0.79 | -0.26 | 0.36 | 1.78 | 72260 | 1 |
| chi_raw[17] | -0.27 | 0.00 | 0.97 | -2.09 | -0.91 | -0.31 | 0.35 | 1.72 | 90506 | 1 |
| chi_raw[18] | 0.14 | 0.00 | 0.92 | -1.65 | -0.47 | 0.12 | 0.73 | 1.99 | 103628 | 1 |
| chi_raw[19] | 0.02 | 0.00 | 1.00 | -1.96 | -0.64 | 0.04 | 0.69 | 1.97 | 96903 | 1 |
| chi_raw[20] | 0.13 | 0.00 | 1.01 | -1.89 | -0.55 | 0.16 | 0.81 | 2.06 | 89012 | 1 |
| chi_0_raw[1] | -0.01 | 0.00 | 1.00 | -1.94 | -0.68 | -0.02 | 0.66 | 1.98 | 112247 | 1 |
| chi_0_raw[2] | 0.04 | 0.00 | 0.97 | -1.86 | -0.62 | 0.03 | 0.68 | 1.96 | 108181 | 1 |
| chi_0_raw[3] | 0.10 | 0.00 | 0.99 | -1.85 | -0.56 | 0.11 | 0.76 | 2.03 | 119537 | 1 |
| chi_0_raw[4] | -0.16 | 0.00 | 0.98 | -2.07 | -0.83 | -0.18 | 0.49 | 1.81 | 105000 | 1 |
| chi_0_raw[5] | 0.04 | 0.00 | 0.98 | -1.85 | -0.63 | 0.03 | 0.70 | 1.99 | 80376 | 1 |
| chi_0_raw[6] | -0.25 | 0.00 | 0.97 | -2.12 | -0.89 | -0.26 | 0.40 | 1.69 | 89514 | 1 |
| chi_0_raw[7] | 0.23 | 0.00 | 0.92 | -1.59 | -0.38 | 0.22 | 0.84 | 2.05 | 89698 | 1 |
| chi_0_raw[8] | -0.02 | 0.00 | 0.96 | -1.92 | -0.66 | -0.01 | 0.63 | 1.88 | 103310 | 1 |
| chi_0_raw[9] | 0.20 | 0.00 | 1.01 | -1.79 | -0.48 | 0.20 | 0.88 | 2.17 | 104757 | 1 |
| chi_0_raw[10] | 0.15 | 0.00 | 0.92 | -1.68 | -0.44 | 0.16 | 0.75 | 1.96 | 95779 | 1 |
| chi_0_raw[11] | 0.00 | 0.00 | 0.96 | -1.87 | -0.65 | -0.01 | 0.65 | 1.92 | 102712 | 1 |
| chi_0_raw[12] | 0.22 | 0.00 | 0.97 | -1.70 | -0.43 | 0.22 | 0.87 | 2.11 | 105415 | 1 |
| chi_0_raw[13] | -0.02 | 0.00 | 1.03 | -2.04 | -0.72 | -0.02 | 0.68 | 1.99 | 97813 | 1 |
| chi_0_raw[14] | 0.06 | 0.00 | 0.97 | -1.84 | -0.58 | 0.06 | 0.71 | 1.96 | 120391 | 1 |
| chi_0_raw[15] | 0.00 | 0.00 | 1.01 | -1.98 | -0.68 | 0.00 | 0.67 | 1.96 | 105015 | 1 |
| chi_0_raw[16] | 0.10 | 0.00 | 0.98 | -1.84 | -0.55 | 0.11 | 0.75 | 2.00 | 120317 | 1 |
| chi_0_raw[17] | -0.14 | 0.00 | 1.00 | -2.07 | -0.81 | -0.15 | 0.53 | 1.85 | 114798 | 1 |
| chi_0_raw[18] | 0.22 | 0.00 | 0.95 | -1.66 | -0.42 | 0.22 | 0.85 | 2.06 | 99796 | 1 |
| chi_0_raw[19] | -0.01 | 0.00 | 1.01 | -1.97 | -0.69 | -0.01 | 0.67 | 1.95 | 129570 | 1 |
| chi_0_raw[20] | 0.03 | 0.00 | 1.00 | -1.94 | -0.65 | 0.03 | 0.71 | 1.99 | 134403 | 1 |
| chi_top | -0.89 | 0.01 | 1.30 | -3.22 | -1.71 | -1.01 | -0.22 | 2.14 | 40059 | 1 |
| chi_0_top | 2.48 | 0.01 | 1.08 | 0.65 | 1.75 | 2.38 | 3.12 | 4.86 | 45968 | 1 |
| chi_sigma_top | 3.31 | 0.11 | 8.87 | 0.17 | 1.24 | 2.26 | 3.90 | 11.75 | 6713 | 1 |
| chi_0_sigma_top | 1.38 | 0.05 | 5.50 | 0.04 | 0.39 | 0.86 | 1.59 | 4.98 | 12833 | 1 |
| chi[1] | -0.04 | 0.05 | 6.44 | -7.69 | -1.98 | -0.46 | 1.26 | 10.08 | 15927 | 1 |
| chi[2] | -0.91 | 0.05 | 6.15 | -8.78 | -2.63 | -1.20 | 0.42 | 9.02 | 14616 | 1 |
| chi[3] | -0.11 | 0.03 | 5.46 | -6.85 | -2.08 | -0.74 | 1.12 | 10.15 | 44775 | 1 |
| chi[4] | -1.85 | 0.11 | 9.68 | -9.68 | -3.24 | -1.69 | -0.35 | 6.74 | 7901 | 1 |
| chi[5] | 1.03 | 0.07 | 6.98 | -3.90 | -1.15 | 0.04 | 1.91 | 11.24 | 10790 | 1 |
| chi[6] | -3.84 | 0.03 | 5.02 | -13.29 | -5.41 | -3.08 | -1.50 | 2.29 | 31830 | 1 |
| chi[7] | -1.80 | 0.12 | 11.46 | -10.34 | -3.22 | -1.85 | -0.69 | 6.64 | 8918 | 1 |
| chi[8] | -2.38 | 0.13 | 10.52 | -9.78 | -3.41 | -2.18 | -1.23 | 3.76 | 6486 | 1 |
| chi[9] | 1.37 | 0.02 | 4.86 | -3.50 | -0.72 | 0.47 | 2.28 | 11.63 | 45660 | 1 |
| chi[10] | -2.39 | 0.13 | 10.30 | -9.02 | -3.57 | -2.30 | -1.39 | 3.81 | 6561 | 1 |
| chi[11] | -2.04 | 0.04 | 6.70 | -11.66 | -3.55 | -1.73 | -0.30 | 6.36 | 27475 | 1 |
| chi[12] | 0.65 | 0.18 | 14.60 | -4.08 | -1.36 | -0.15 | 1.78 | 11.40 | 6765 | 1 |
| chi[13] | 0.13 | 0.15 | 11.99 | -6.21 | -1.93 | -0.62 | 1.32 | 9.98 | 6800 | 1 |
| chi[14] | -0.49 | 0.21 | 16.51 | -7.90 | -2.33 | -1.09 | 0.41 | 8.85 | 6082 | 1 |
| chi[15] | -0.17 | 0.07 | 7.47 | -6.21 | -2.09 | -0.83 | 0.90 | 9.78 | 10806 | 1 |
| chi[16] | -1.28 | 0.14 | 12.97 | -8.46 | -2.93 | -1.68 | -0.42 | 7.58 | 8410 | 1 |
| chi[17] | -2.04 | 0.09 | 8.64 | -11.27 | -3.34 | -1.72 | -0.39 | 6.40 | 9438 | 1 |
| chi[18] | 0.05 | 0.19 | 15.39 | -5.64 | -2.11 | -0.88 | 0.86 | 10.14 | 6769 | 1 |
| chi[19] | -0.84 | 0.05 | 6.18 | -9.21 | -2.48 | -1.05 | 0.60 | 8.82 | 18257 | 1 |
| chi[20] | -0.46 | 0.03 | 5.38 | -8.54 | -2.28 | -0.79 | 1.02 | 9.45 | 37170 | 1 |
| chi_0[1] | 2.53 | 0.02 | 3.65 | -1.12 | 1.50 | 2.36 | 3.39 | 6.98 | 38656 | 1 |
| chi_0[2] | 2.67 | 0.04 | 5.35 | -0.40 | 1.58 | 2.40 | 3.41 | 6.93 | 15689 | 1 |
| chi_0[3] | 2.69 | 0.04 | 5.42 | -0.58 | 1.64 | 2.49 | 3.55 | 7.26 | 15945 | 1 |
| chi_0[4] | 2.18 | 0.04 | 5.65 | -1.58 | 1.33 | 2.18 | 3.12 | 6.05 | 18664 | 1 |
| chi_0[5] | 2.85 | 0.07 | 7.98 | 0.02 | 1.54 | 2.37 | 3.46 | 7.55 | 14768 | 1 |
| chi_0[6] | 1.97 | 0.05 | 6.78 | -1.86 | 1.28 | 2.10 | 3.01 | 5.53 | 17331 | 1 |
| chi_0[7] | 3.07 | 0.04 | 5.27 | 0.74 | 1.85 | 2.60 | 3.62 | 7.45 | 15369 | 1 |
| chi_0[8] | 2.38 | 0.03 | 4.16 | -1.18 | 1.54 | 2.36 | 3.34 | 6.26 | 26541 | 1 |
| chi_0[9] | 3.04 | 0.10 | 11.09 | -0.38 | 1.72 | 2.59 | 3.71 | 8.00 | 12405 | 1 |
| chi_0[10] | 2.76 | 0.05 | 6.55 | 0.38 | 1.79 | 2.55 | 3.51 | 6.65 | 17508 | 1 |
| chi_0[11] | 2.63 | 0.02 | 3.32 | -0.23 | 1.55 | 2.35 | 3.36 | 6.79 | 33716 | 1 |
| chi_0[12] | 3.02 | 0.03 | 4.60 | 0.03 | 1.78 | 2.62 | 3.71 | 7.69 | 26738 | 1 |
| chi_0[13] | 2.43 | 0.02 | 3.46 | -1.93 | 1.44 | 2.35 | 3.41 | 6.83 | 32528 | 1 |
| chi_0[14] | 2.71 | 0.11 | 11.70 | -0.72 | 1.61 | 2.44 | 3.45 | 6.81 | 11341 | 1 |
| chi_0[15] | 2.41 | 0.09 | 9.67 | -1.43 | 1.51 | 2.37 | 3.41 | 6.82 | 11509 | 1 |
| chi_0[16] | 2.72 | 0.06 | 7.31 | -0.67 | 1.65 | 2.49 | 3.55 | 6.96 | 13659 | 1 |
| chi_0[17] | 2.12 | 0.08 | 9.22 | -1.87 | 1.33 | 2.21 | 3.20 | 6.13 | 12509 | 1 |
| chi_0[18] | 3.00 | 0.02 | 3.55 | 0.30 | 1.80 | 2.61 | 3.68 | 7.60 | 24391 | 1 |
| chi_0[19] | 2.43 | 0.03 | 5.03 | -1.47 | 1.49 | 2.36 | 3.39 | 6.78 | 31021 | 1 |
| chi_0[20] | 2.49 | 0.05 | 6.11 | -1.26 | 1.54 | 2.39 | 3.44 | 6.96 | 15850 | 1 |
| delta_0[1] | 1.11 | 0.00 | 0.14 | 0.85 | 1.02 | 1.11 | 1.20 | 1.39 | 38519 | 1 |
| delta_0[2] | 1.05 | 0.00 | 0.11 | 0.83 | 0.98 | 1.05 | 1.12 | 1.26 | 65449 | 1 |
| delta_0[3] | 0.84 | 0.00 | 0.11 | 0.61 | 0.76 | 0.84 | 0.91 | 1.06 | 54222 | 1 |
| delta_0[4] | 0.91 | 0.00 | 0.10 | 0.71 | 0.84 | 0.91 | 0.98 | 1.11 | 62595 | 1 |
| delta_0[5] | 0.97 | 0.00 | 0.11 | 0.72 | 0.90 | 0.97 | 1.04 | 1.18 | 47977 | 1 |
| delta_0[6] | 0.99 | 0.00 | 0.10 | 0.79 | 0.92 | 0.99 | 1.05 | 1.18 | 67482 | 1 |
| delta_0[7] | 1.07 | 0.00 | 0.10 | 0.86 | 1.00 | 1.07 | 1.14 | 1.27 | 66891 | 1 |
| delta_0[8] | 0.92 | 0.00 | 0.13 | 0.67 | 0.83 | 0.92 | 1.01 | 1.18 | 51960 | 1 |
| delta_0[9] | 0.80 | 0.00 | 0.15 | 0.52 | 0.70 | 0.79 | 0.89 | 1.10 | 37268 | 1 |
| delta_0[10] | 1.25 | 0.00 | 0.11 | 1.03 | 1.18 | 1.25 | 1.33 | 1.47 | 57371 | 1 |
| delta_0[11] | 1.10 | 0.00 | 0.10 | 0.91 | 1.04 | 1.10 | 1.17 | 1.29 | 75280 | 1 |
| delta_0[12] | 0.92 | 0.00 | 0.10 | 0.72 | 0.85 | 0.92 | 0.98 | 1.11 | 76945 | 1 |
| delta_0[13] | 0.89 | 0.00 | 0.10 | 0.70 | 0.83 | 0.89 | 0.96 | 1.09 | 58647 | 1 |
| delta_0[14] | 1.21 | 0.00 | 0.10 | 1.02 | 1.14 | 1.21 | 1.27 | 1.40 | 77060 | 1 |
| delta_0[15] | 1.14 | 0.00 | 0.10 | 0.93 | 1.07 | 1.14 | 1.21 | 1.33 | 70843 | 1 |
| delta_0[16] | 1.09 | 0.00 | 0.11 | 0.87 | 1.01 | 1.08 | 1.16 | 1.31 | 68355 | 1 |
| delta_0[17] | 0.97 | 0.00 | 0.10 | 0.78 | 0.91 | 0.97 | 1.04 | 1.17 | 79017 | 1 |
| delta_0[18] | 1.12 | 0.00 | 0.10 | 0.94 | 1.06 | 1.12 | 1.19 | 1.32 | 68892 | 1 |
| delta_0[19] | 0.81 | 0.00 | 0.12 | 0.59 | 0.73 | 0.81 | 0.89 | 1.04 | 55257 | 1 |
| delta_0[20] | 1.19 | 0.00 | 0.14 | 0.91 | 1.10 | 1.19 | 1.28 | 1.47 | 56424 | 1 |
| eta[1] | 0.05 | 0.00 | 0.05 | -0.04 | 0.02 | 0.05 | 0.08 | 0.17 | 42267 | 1 |
| eta[2] | 0.02 | 0.00 | 0.04 | -0.06 | -0.01 | 0.02 | 0.05 | 0.11 | 73428 | 1 |
| eta[3] | 0.00 | 0.00 | 0.04 | -0.07 | -0.02 | 0.00 | 0.03 | 0.08 | 58268 | 1 |
| eta[4] | 0.01 | 0.00 | 0.06 | -0.13 | -0.03 | 0.01 | 0.05 | 0.14 | 61890 | 1 |
| eta[5] | -0.01 | 0.00 | 0.06 | -0.15 | -0.05 | 0.00 | 0.03 | 0.10 | 50086 | 1 |
| eta[6] | 0.04 | 0.00 | 0.08 | -0.09 | -0.01 | 0.03 | 0.07 | 0.22 | 48523 | 1 |
| eta[7] | 0.00 | 0.00 | 0.06 | -0.13 | -0.03 | 0.00 | 0.04 | 0.12 | 50670 | 1 |
| eta[8] | 0.02 | 0.00 | 0.07 | -0.11 | -0.02 | 0.02 | 0.06 | 0.17 | 54056 | 1 |
| eta[9] | -0.02 | 0.00 | 0.06 | -0.15 | -0.05 | -0.01 | 0.02 | 0.08 | 32032 | 1 |
| eta[10] | 0.01 | 0.00 | 0.07 | -0.15 | -0.03 | 0.01 | 0.05 | 0.13 | 43162 | 1 |
| eta[11] | 0.03 | 0.00 | 0.07 | -0.10 | -0.01 | 0.02 | 0.07 | 0.20 | 59597 | 1 |
| eta[12] | 0.02 | 0.00 | 0.06 | -0.09 | -0.01 | 0.02 | 0.06 | 0.15 | 67419 | 1 |
| eta[13] | 0.03 | 0.00 | 0.07 | -0.11 | -0.01 | 0.02 | 0.06 | 0.19 | 65940 | 1 |
| eta[14] | 0.03 | 0.00 | 0.06 | -0.10 | -0.01 | 0.02 | 0.06 | 0.16 | 57796 | 1 |
| eta[15] | 0.02 | 0.00 | 0.07 | -0.12 | -0.02 | 0.02 | 0.05 | 0.16 | 73611 | 1 |
| eta[16] | -0.01 | 0.00 | 0.06 | -0.14 | -0.05 | -0.01 | 0.03 | 0.09 | 45576 | 1 |
| eta[17] | 0.03 | 0.00 | 0.06 | -0.08 | -0.01 | 0.03 | 0.06 | 0.16 | 58919 | 1 |
| eta[18] | 0.01 | 0.00 | 0.06 | -0.12 | -0.02 | 0.01 | 0.05 | 0.15 | 77554 | 1 |
| eta[19] | 0.00 | 0.00 | 0.06 | -0.15 | -0.04 | 0.00 | 0.04 | 0.11 | 43586 | 1 |
| eta[20] | 0.03 | 0.00 | 0.04 | -0.06 | 0.00 | 0.02 | 0.05 | 0.11 | 57414 | 1 |
| delta_0_av | 1.00 | 0.00 | 0.20 | 0.60 | 0.88 | 1.00 | 1.12 | 1.38 | 82127 | 1 |
| eta_av | 0.01 | 0.00 | 0.07 | -0.13 | -0.02 | 0.01 | 0.05 | 0.16 | 73649 | 1 |
| chi_av | -0.94 | 0.03 | 9.76 | -9.34 | -2.61 | -1.13 | 0.56 | 8.74 | 81674 | 1 |
| chi_0_av | 2.47 | 0.02 | 6.20 | -1.45 | 1.51 | 2.37 | 3.39 | 6.78 | 118659 | 1 |
| lp__ | 485.04 | 0.06 | 10.18 | 464.12 | 478.37 | 485.40 | 492.07 | 503.98 | 26881 | 1 |

Samples were drawn using NUTS(diag_e) at Tue Apr 30 21:48:05 2019.

For each parameter, n_eff is a crude measure of effective sample size,

and Rhat is the potential scale reduction factor on split chains (at

convergence, Rhat=1).
